# Dephosphorylation accelerates the dissociation of ZAP70 from the T cell receptor

**DOI:** 10.1073/pnas.2116815119

**Published:** 2022-02-23

**Authors:** Jesse Goyette, David Depoil, Zhengmin Yang, Samuel A. Isaacson, Jun Allard, P. Anton van der Merwe, Katharina Gaus, Michael L. Dustin, Omer Dushek

**Affiliations:** ^a^European Molecular Biology Laboratory Australia Node in Single Molecule Science, School of Medical Sciences, University of New South Wales, Sydney 2052, NSW, Australia;; ^b^Australian Research Council Centre of Excellence in Advanced Molecular Imaging, University of New South Wales, Sydney 2052, NSW, Australia;; ^c^The Kennedy Institute of Rheumatology, University of Oxford, OX3 7FY Oxford, United Kingdom;; ^d^Department of Mathematics and Statistics, Boston University, Boston, MA 02215;; ^e^Center for Complex Biological Systems, University of California, Irvine, CA 92697;; ^f^Sir William Dunn School of Pathology, University of Oxford, OX1 3RE Oxford, United Kingdom

**Keywords:** T cell receptor, signal transduction, kinetic proofreading, signal reversibility, antigen discrimination

## Abstract

Protein–protein binding domains are critical in signaling networks. Src homology 2 (SH2) domains are binding domains that interact with sequences containing phosphorylated tyrosines. A subset of SH2 domain–containing proteins has tandem domains, which are thought to enhance binding affinity and specificity. However, a trade-off exists between long-lived binding and the ability to rapidly reverse signaling, which is a critical requirement of noise-filtering mechanisms such as kinetic proofreading. Here, we use modeling to show that the unbinding rate of tandem, but not single, SH2 domains can be accelerated by phosphatases. Using surface plasmon resonance, we show that the phosphatase CD45 can accelerate the unbinding rate of zeta chain–associated protein kinase 70 (ZAP70), a tandem SH2 domain–containing kinase, from biphosphorylated peptides from the T cell receptor (TCR). An important functional prediction of accelerated unbinding is that the intracellular ZAP70–TCR half-life in T cells will not be fixed but rather, dependent on the extracellular TCR–antigen half-life, and we show that this is the case in both cell lines and primary T cells. The work highlights that tandem SH2 domains can break the trade-off between signal fidelity (requiring long half-life) and signal reversibility (requiring short half-life), which is a key requirement for T cell antigen discrimination mediated by kinetic proofreading.

## Significance

Src homology 2 (SH2) domains are phosphotyrosine binding motifs that play key roles in cellular signaling. There are 110 proteins in the human genome containing SH2 binding domains, of which 10 contain tandem SH2 domains. Tandem domains have been shown to improve avidity and specificity and contribute to allostery. Here, we show that tandem SH2 domains can also exhibit binding lifetimes that are accelerated by the activity of phosphatases. This accelerated unbinding requires tandem SH2 domains to engage their substrates in dynamic binding modes that cycle between single SH2-bound states. We experimentally confirm that this is the case for the well-studied kinase ZAP70 binding the T cell receptor. We suggest that accelerated unbinding is a general feature of signaling networks.

Protein–protein binding domains are fundamental to signaling networks (1–3). Many binding domains recognize posttranslational modifications; an archetypal example is the Src homology 2 (SH2) domain, which binds to phosphorylated tyrosines within disordered regions of proteins (4). SH2 domain–containing proteins are critical for signaling downstream of many surface receptors that become phosphorylated on their cytoplasmic tails upon ligand binding [e.g., receptor tyrosine kinases (5) and noncatalytic tyrosine-phosphorylated receptors (NTRs) (6)]. Two well-studied protein families that contain SH2 domains are Src and spleen tyrosine kinase (Syk) kinases, and in both families, SH2 domains are implicated in localization and allosteric activation (7–10). Of the 110 proteins in humans with SH2 domains, 100 contain a single SH2 domain (e.g., Src kinases), but only 10 contain tandem SH2 domains (e.g., Syk kinases). The precise function of tandem SH2 domains is unclear.

The Syk family contains two cytosolic proteins, Syk and ZAP70 (zeta chain–associated protein kinase 70), and both have tandem SH2 domains that bind biphosphorylated immunotyrosine-based activation motifs (ITAMs; YxxL/Ix${}_{6-8}$ YxxL/I) that are found on the cytoplasmic tails of activating NTRs, such as Fc receptors, B cell receptors, and T cell receptors (TCRs) (6). Binding of tandem SH2 domains to biphosphorylated ITAMs is thought to improve specificity (11), increase affinity (12–14), and/or induce structural allosteric activation of the kinase domain (15–19). However, these functions are not unique to tandem SH2 domains, raising the question of whether tandem domains simply have quantitative advantages over single SH2 domains or whether they can exhibit qualitatively distinct behaviors.

An often overlooked property of signaling networks is the mechanism(s) by which they can be efficiently reversed. This property is important for the ability of T cells to appropriately initiate and regulate adaptive immunity to typically rare foreign antigens while ignoring the far more abundant self-antigens. They do this by discriminating between ligands, usually peptides presented on major histocompatibility complexes (pMHCs) based on their binding affinity or half-life for the TCR (20, 21). The mechanism that can explain this discrimination is called kinetic proofreading (22–30). This mechanism postulates that the binding of a pMHC to the TCR initiates a sequence of biochemical steps that introduce a delay between binding and productive TCR signaling. At a molecular level, these steps may include the phosphorylation of ITAMs by the Src family kinase Lck and the subsequent recruitment and activation of ZAP70, which activates downstream signaling pathways (10). Because of the requirement for multiple time-consuming steps, productive TCR signaling can be highly dependent on the TCR–pMHC half-life. Critically, kinetic proofreading requires that the signaling steps are rapidly reversed upon TCR–pMHC unbinding. Without rapid reversal, productive TCR signaling could be initiated by sequential short-lived interactions of the TCR with self-pMHC. Ligand binding to TCR has been proposed to initiate TCR signaling by spatially segregating it from the inhibitory receptor phosphatase CD45 (31). This requires that CD45 can reverse phosphorylation of the TCR–CD3 complex. However, biochemical studies show that ZAP70 binds with high affinity to phosphorylated TCR ITAMs (12, 32, 33), which would shield these phosphorylation sites from CD45 activity (34). This highlights an affinity trade-off with SH2 domain interactions; increasing affinity can strengthen signaling, but by preventing receptor dephosphorylation, they could decrease discrimination by allowing receptors to sustain signaling even after ligand unbinding.

Here, we demonstrate that ZAP70 recruitment compromises kinetic proofreading unless its dissociation can be selectively accelerated by phosphatases after TCR–pMHC unbinding. Using modeling and experiments, we show that the long half-life of ZAP70 is achieved by its SH2 domains continually unbinding and rebinding individual phosphorylated tyrosines in the ITAM and that the phosphatase CD45 can access these tyrosines to accelerate the dissociation of ZAP70. A key prediction of accelerated unbinding is that the in vivo intracellular ZAP70–ITAM binding half-life is not a fixed quantity but rather, that it is coupled to how long the TCR remains bound to pMHC, and we confirm that this is the case using live cell microscopy in T cells. The work highlights that tandem SH2 domains can break the trade-off between signal robustness (requiring a long half-life) and signal reversibility (requiring a short half-life) to faithfully couple TCR–pMHC binding with TCR signaling, a key requirement for kinetic proofreading.

## Results

### Regulated Unbinding of ZAP70 Can Maintain Kinetic Proofreading at the TCR.

**Fig. 1.**
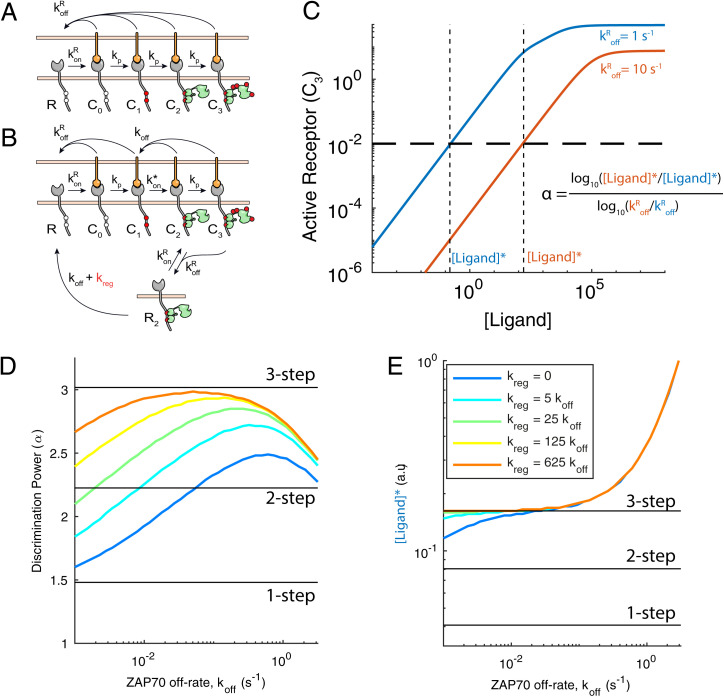
The operational model shows that ZAP70 unbinding impairs kinetic proofreading unless it is regulated. (*A*) Standard three-step kinetic proofreading showing that pMHC binding to the TCR initiates a sequence of steps—1) ITAM phosphorylation, 2) ZAP70 recruitment, and 3) ZAP70 phosphorylation—that can result in a signaling active TCR (*C*_3_). All steps are assumed to be immediately reversed upon pMHC unbinding ($k_{\text{off}}^{\text{R}}$). (*B*) Modified kinetic proofreading that explicitly models ZAP70 recruitment showing that ZAP70 can bind and unbind to phosphorylated TCR when it is bound to pMHC (with rate $k_{\text{on}}^{*}$ and *k*_off)_ and that ZAP70 can remain bound after pMHC unbinding (*R*_2_). The model assumes an excess of ZAP70 in the cytosol so that binding is a first-order rate. Regulated unbinding ($k_{\text{reg}}$) selectively accelerates unbinding of ZAP70 when the TCR is unbound (state *R*_2_). (*C*) Example dose–response curves show the concentration of productively signaling TCRs (*C*_3_) over the ligand concentration for higher-affinity (blue) and lower-affinity (red) ligands. The horizontal dashed line marks the arbitrary threshold concentration of productively signaling TCRs required to activate T cells, and vertical dashed lines mark the concentration of each ligand required to achieve this threshold. (*D* and *E*) Calculations showing (*D*) the discriminatory power and (*E*) sensitivity over the ZAP70 off-rate for the indicated values of the regulated off-rate $k_{\text{reg}}$ (colors). The discriminatory power (*α*) is calculated using the formula in *C*, and sensitivity is the concentration of the higher-affinity ligand required to activate T cells. Horizontal solid black lines in *D* and *E* are results for the standard proofreading model with the indicated number of steps.

The kinetic proofreading model can explain the ability of the TCR to discriminate pMHC ligands based on their half-life or unbinding rate from the TCR ($k_{\text{off}}^{\text{R}}$). It achieves this by introducing a time delay between pMHC binding and productive TCR signaling by requiring multiple biochemical steps (Fig. 1*A*). Importantly, the model assumes that all biochemical steps are immediately reversed when the pMHC unbinds from the TCR. This ensures that the TCR does not sustain any signals by binding repeatedly to pMHCs with faster off-rates. Modifying kinetic proofreading to explicitly include ZAP70 recruitment introduces a sustained signaling state, whereby ZAP70 can remain bound to the TCR even after pMHC unbinding (Fig. 1*B*, *R*_2_).

To explore the impact of introducing sustained signaling, we quantified its effect on antigen discrimination and sensitivity using mathematical modeling. We first calculated the concentration of productive signaling TCR complexes (*C*_3_) using two test pMHC ligands with a 10-fold difference in their unbinding rate (Fig. 1*C*). We defined a threshold amount of productively signaling TCRs required to activate a T cell (Fig. 1*C*, horizontal line), which allowed us to calculate the concentration of each ligand required to achieve this activation threshold ([Ligand]*). The ability of the TCR to discriminate between these ligands can then be quantified by a discriminatory power (*α*) defined as the fold change in [Ligand]* over the fold change in their unbinding rates. A large discriminatory power corresponds to a larger fold change in [Ligand]* between the two ligands, which enables the T cell to distinguish between these two ligands over a wider concentration range. The antigen sensitivity of T cells can be quantified by [Ligand]* of the higher-affinity ligand, with smaller values corresponding to higher sensitivity.

Using the standard kinetic proofreading model, we confirmed previous results showing that increasing the number of steps from one to three increased the discriminatory power but decreased antigen sensitivity (Fig. 1 *D* and *E*, horizontal lines). Next, we used the modified model that explicitly included ZAP70 recruitment and systematically varied the ZAP70 off-rate (*k*_off)_ (Fig. 1 *C* and *D*, blue line with $k_{\text{reg}}=0$). We found that when the ZAP70 off-rate was decreased, the discriminatory power decreased and approached the value obtained with the one-step kinetic proofreading model (Fig. 1*D*). This was a consequence of ZAP70 remaining bound to the TCR after pMHC unbinding, allowing pMHCs with shorter half-lives to bypass earlier steps. On the other hand, when the ZAP70 *k*_off _was increased to levels that improved discrimination, TCR signaling required high concentrations of ligand (Fig. 1*E*). Hence, there is a trade-off between sensitivity, which improves as the ZAP70 *k*_off _decreases, and discrimination, which improves as *k*_off _increases.

We next explored how discrimination and sensitivity could be optimized. While the TCR–CD3 complex is segregated from CD45 when it is bound to pMHC, it is likely to be exposed to CD45 soon after dissociating. We suggest that CD45 accelerates ZAP70 dissociation from the TCR, which we term regulated unbinding. We introduced regulated unbinding (Fig. 1*B*, with rate $k_{\text{reg}}$ accelerating the transition from *R*_2_ to *R*) and found that the proofreading chain can be restored with improved discrimination when $k_{\text{off}}<$ 1 s^−1^ (Fig. 1*D*) and a negligible impact on sensitivity (Fig. 1*E*). Therefore, the trade-off between sensitivity and discrimination can be reduced by regulation of ZAP70 unbinding.

### Dynamic Internal States of the ZAP70–ITAM Complex Support a Phosphatase-Regulated Unbinding Mechanism.

**Fig. 2.**
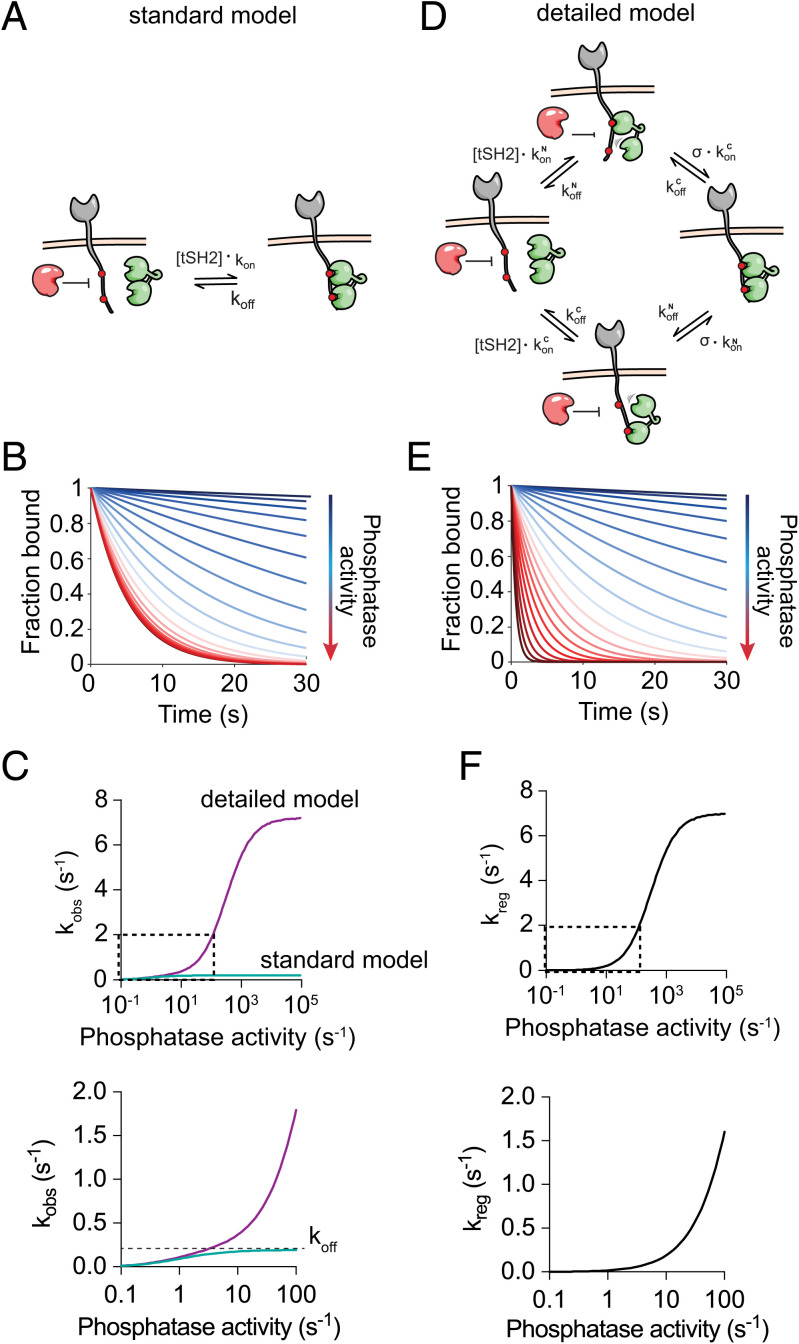
A detailed model of ZAP70 to ITAMs, which incorporates multiple internal states, predicts regulated unbinding by phosphatases. (*A*) A standard 1:1 binding model that assumes that phosphatases (red) can dephosphorylate exposed (nonbound) phosphotyrosines. (*B*) The amount of ZAP70 bound over time for increasing protein tyrosine phosphatase (PTP) activity for the model in *A*. (*C*) The observed unbinding rate over PTP activity determined by exponential fit to the standard (green) and detailed (purple) binding models. (*D*) The detailed binding model for ZAP70 reflecting dynamic internal states of the complex parameterized by an on-rate and an off-rate for each SH2 domain binding and each phosphotyrosine and by the local concentration of phosphotyrosine experienced by the unbound SH2 domain when ZAP70 is bound by the other SH2 domain (*σ*). In this model, phosphotyrosines are exposed even when ZAP70 is bound to the ITAM. (*E*) The amount of ZAP70 bound over time for increasing PTP activity for the model in *D*. (*F*) The regulated unbinding rate is calculated by the difference in observed off-rates between the detailed and standard binding models in *C*. The boxed section in *Upper* is shown enlarged in *Lower*.

We next investigated molecular mechanisms that can support regulated unbinding. The ZAP70–ITAM interaction may be approximated by a 1:1 binding model, which also describes single SH2 domains, if ZAP70 spends the majority of its time simultaneously engaged with both SH2 domains (Fig. 2*A*). We refer to this mode of binding as the standard model, in which binding is characterized by a single dominant state. We used this model to calculate the amount of bound ZAP70 over time with different phosphatase activities in the presence of a constant concentration of solution ZAP70 that can continuously bind and unbind to phosphorylated ITAMs (Fig. 2*B*). The observed unbinding rate (i.e., the rate at which ZAP70 occupation of ITAMs decays to zero) increased with increasing phosphatase activity but was limited to the ZAP70 off-rate since phosphatases cannot dephosphorylate ITAMs bound by SH2 domains (Fig. 2*C*).

We next constructed a detailed ZAP70 binding model (Fig. 2*D*). In this model, we explicitly included the initial interaction of each SH2 domain and the subsequent interaction of the second domain mediated by intrinsic on-rates that are dependent on the effective concentration of free phosphotyrosine (which we refer to as *σ*, in units of micromolar). Using model parameters that allowed ZAP70 to rapidly cycle between these internal states yet achieve the same overall unbinding rate used in the standard 1:1 model above, we found that the observed off-rate could exceed the ZAP70 off-rate as the phosphatase activity was increased (Fig. 2 *C* and *E*). In order to quantify the contribution of phosphatase activity to the accelerated dissociation of ZAP70, we plotted the difference in observed off-rates between the two models, which showed that this regulated unbinding rate increases with phosphatase activity (Fig. 2*F*). Therefore, the unbinding of ZAP70 can be accelerated by phosphatases. Importantly, this requires that the phosphatase is not sterically blocked from accessing an exposed ITAM phosphotyrosine when ZAP70 is bound to the other ITAM phosphotyrosine.

### Systematic Analysis Suggests That ZAP70 Binding to ITAM Involves Dynamic Internal States.

**Fig. 3.**
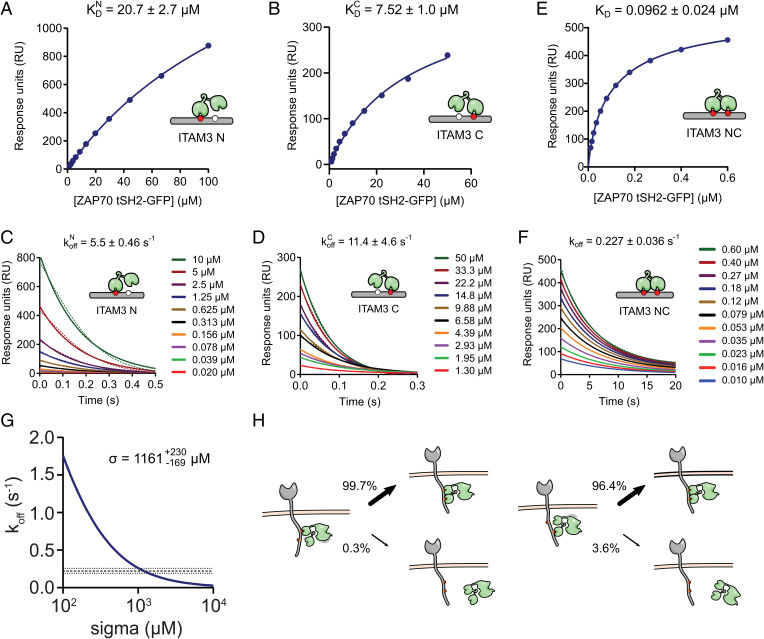
SPR binding affinity and kinetics of ZAP70 interacting with mono- and biphosphorylated ITAM peptides parameterize the mathematical model and predict dynamic internal states of the ZAP70–ITAM complex. Representative equilibrium and dissociation phase data from the tSH2-GFP protein interacting with (*A* and *C*) N- or (*B* and *D*) C-monophosphorylated or (*E* and *F*) biphosphorylated CD3*ζ* ITAM3 peptides. Indicated parameters are obtained by fitting the data (dots) with a 1:1 binding model (solid lines). (*G*) Unbinding rates of ZAP70 from biphosphorylated ITAM (*y* axis) were calculated using the model for different values of *σ* (*x* axis) with the four kinetic rate constants fixed to their experimentally determined values (Table 1). The mean (dashed line) ± SEM (dotted lines) of the bivalent dissociation rate is shown, and it intersects at the indicated value of *σ*. (*H*) Probability of rebinding or unbinding when ZAP70 is bound to N-terminal (*Left*) or C-terminal (*Right*) tyrosines of ITAM3 using the value of *σ* determined in *G* (the text has the calculation) predicts that the long half-life of ZAP70 is achieved by kinetically cycling between states bound by one and both SH2 domains.

To determine the binding mode of ZAP70, we experimentally determined all model parameters. To do this, we used the tandem Src homology 2 domains of ZAP70 (tSH2s) and bi- or monophosphorylated ITAM peptides derived from the membrane-distal immunotyrosine-based activation motif of CD3*ζ* (ITAM3). As expected, the 1:1 binding model was able to describe binding of tSH2 to monophosphorylated ITAM3 N and C peptides at steady state (Fig. 3 *A* and *B*). A kinetic analysis showed that on-rates were not measurable by surface plasmon resonance (SPR) due to mass transport; however, estimates of the off-rates were possible, revealing rapid unbinding (Fig. 3 *C* and *D*). Therefore, on-rates were determined using off-rates and dissociation constants (Table 1).

**Table 1.** Affinity and kinetic measurements of ZAP70 on ITAM3 peptides by SPR at 37 ${}^{{}^{\circ}}$C (*N* ≥ 3)

Peptide	*K*_D _(µM)	$k_{\text{on}}$ (µM^–1^ s^–1^)	*k*_off _(s^–1^)	${\text{t}}_{\text{1/2}}$ (s)	*n*
ITAM3 N	20.7 ± 2.7	0.265*	5.5 ± 0.46	0.126	4
ITAM3 C	7.52 ± 1.0	1.52*	11.4 ± 4.6	0.061	4
ITAM3 NC	0.0962 ± 0.024	1.89 ± 0.356	0.227 ± 0.036	3.05	6
					

*Values are estimated from measured *K*_D _and *k*_off._ Parameter values are means of fits to individual SPR experiments ± SEM. Numbers of SPR experiments for each condition are shown in the column labeled *n*.

The interaction between tSH2 and biphosphorylated ITAM peptides was also well described by a 1:1 binding model revealing an effective *K*_D _of 0.096 µM (Fig. 3*E*), *k*_off _of 0.227 s^−1^ (Fig. 3*F*), and $k_{\text{on}}$ of 1.89 µM^−1^ s^−1^ (*SI Appendix*, Fig. S1). This was predicted by the bivalent model, which produced a binding equation that was identical in form to the monomeric model except that the fitted *K*_D _value was dependent on the individual reaction parameters: $K_{\text{D}}=K_{\text{D}}^{\text{N}}K_{\text{D}}^{\text{C}}/(\sigma +K_{\text{D}}^{\text{N}}+K_{\text{D}}^{\text{C}})$ (*Materials and Methods*). Given that all dissociation constants were determined, it was possible to use this equation to directly calculate *σ* to be 1,733 ± 743 µM. We also estimated *σ* directly from the kinetic data by calculating the ZAP70 off-rate for different values of *σ*, fixing the four kinetic rate constants to their experimentally determined values. As expected, the predicted ZAP70 off-rate decreased when *σ* increased with the experimentally determined off-rate obtained when *σ* was 1,161 µM (Fig. 3*G*). This was within the error of the value determined above and similar to previous estimates for Syk (35, 36). Given that *k*_off _is estimated with higher accuracy compared with *K*_D _in SPR, we proceeded to use the estimate of *σ* obtained by the kinetic data.

These rate constants suggest that the long half-life of ZAP70 on biphosphorylated ITAMs (3.05 s) is achieved by cycles of unbinding and rebinding of individual SH2 domains, which have very short half-lives (0.126 and 0.061 s). Indeed, using our estimate for *σ*, we calculated the fraction of time that ZAP70, when bound by a single SH2 domain, would rebind with the other SH2 domains vs. unbinding back to solution [e.g., $P_{\text{rebind}}^{\text{N}}=\sigma k_{\text{on}}^{\text{C}}/(\sigma k_{\text{on}}^{\text{C}}+k_{\text{off}}^{\text{N}})=0.997$, when ZAP70 is bound to ITAM N, and $P_{\text{rebind}}^{\text{C}}=0.964$, when ZAP70 is bound to ITAM C]. These calculations revealed that ZAP70 rebinds over 96% of the time compared with unbinding from the ITAM, showing that the long half-life is maintained by a dynamic binding mode in which ZAP70 cycles between internal binding states (Fig. 3*H*).

The analysis thus far was based on the assumption that the unbinding rate of each SH2 domain is independent of the binding status of the other. However, it has been shown that the SH2 domains of ZAP70 undergo a conformational change when both are simultaneously bound (10). This raises the possibility that ZAP70 achieves the long half-life of 3.05 s by selectively increasing the half-life of each individual SH2 domain when both are bound so that instead of dynamically cycling between internal states, ZAP70 spends the majority of the time in a single state where both SH2 domains are bound. In this case, ZAP70 binding could not be regulated by phosphatases. To explore this possibility, we introduced a cooperativity parameter (*λ*) that multiplied the off-rates of each SH2 domain when both were simultaneously bound (*SI Appendix*, Fig. S2*A*). In this model, we were unable to uniquely determine *λ* and *σ*, but we were able to place bounds on them showing that the largest degree of cooperativity (i.e., smallest value of *λ*) that could explain the data was 0.3; at this value, *σ* was 300 µM (*SI Appendix*, Fig. S2*B*). With these parameters, we found that $P_{\text{rebind}}^{\text{N}}=0.988$ and $P_{\text{rebind}}^{\text{C}}=0.875$ (*SI Appendix*, Fig. S2*C*). This result indicates that even if ZAP70 exhibits binding cooperativity, this effect will not lock the complex in a bivalent SH2-bound state. Instead, ZAP70–ITAM complexes would still cycle through single SH2-bound states, thus exposing phosphorylated tyrosines and allowing binding to be regulated by phosphatases.

### The Phosphatase CD45 Can Accelerate the Dissociation of ZAP70 from ITAMs.

**Fig. 4.**
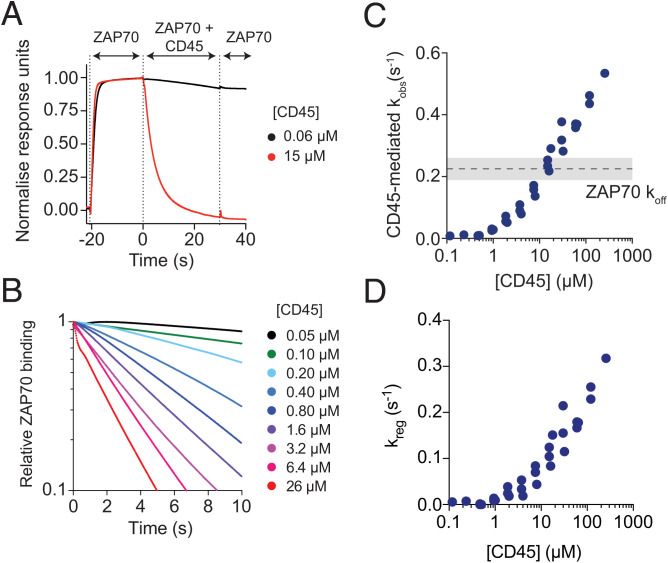
CD45 increases the unbinding rate of ZAP70 from ITAMs beyond the ZAP70 off-rate. (*A*) Example sensogram of the CD45-accelerated ZAP70 unbinding assay in SPR. ZAP70 (500 nM) was first injected over a surface of the ITAM3–phosphorylated CD3*ζ* cytoplasmic domain and allowed to reach steady state before a mixture of ZAP70 (500 nM) and CD45 (indicated concentration) was injected, and finally, ZAP70 (500 nM) was injected. (*B*) The ZAP70 and CD45 coinjection phase for multiple concentrations of CD45 demonstrating a concentration-dependent acceleration in the loss of ZAP70 binding. (*C*) The fitted observed unbinding rate over [CD45] (results from three experiments conducted on different days are shown). The ZAP70 off-rate is shown as a dashed line with ± SEM shaded in gray. (*D*) The regulated off-rate calculated over [CD45]. Binding of ZAP70 to this full-length Avi-CD3*ζ* ITAM3 was the same as on the shorter ITAM3 peptide (*SI Appendix*, Fig. S4).

A key consequence of ZAP70 binding to ITAMs having dynamic internal states is the potential for phosphatases to dephosphorylate exposed ITAM tyrosines and hence, regulate the unbinding of ZAP70 (Fig. 2). This assumes that exposed phosphotyrosines are sterically accessible by phosphatases, which we directly tested using an SPR assay for phosphatase-accelerated unbinding. In this assay, an N-terminally Avitagged, biotinylated, and phosphorylated peptide corresponding to the full intracellular tail of CD3*ζ* with all tyrosines mutated to Ala except the C-terminal ITAM (Avi-CD3*ζ* ITAM3) was immobilized on the chip surface. A near-saturating concentration of ZAP70 tSH2 was injected over the surface and allowed to reach steady state before the same concentration of ZAP70 mixed with different concentrations of the cytoplasmic domain of the phosphatase CD45 was injected (Fig. 4*A*). This assay format allowed us to assess the rate of ITAM dephosphorylation by CD45 in the presence of ZAP70, as would be the case in T cells.

In the presence of CD45, the amount of ZAP70 binding to the chip surface decreased over time (Fig. 4 *A* and *B*). If CD45 only dephosphorylated free ITAMs, then the maximum observed rate of ZAP70 unbinding would be the ZAP70 unbinding rate *k*_off._ However, the results clearly demonstrate that beyond 10 µM CD45, the observed off-rate exceeded *k*_off,_ indicating that CD45 can dephosphorylate ITAMs bound by ZAP70 (Fig. 4 *B* and *C*). As before, we calculated the CD45-mediated regulated off-rate by subtracting the experimental observed off-rate from the predicted observed off-rate in the standard ZAP70–ITAM binding model (Fig. 4*D*). This result was also observed with different immobilization levels of Avi-CD3*ζ* ITAM3 demonstrating that SPR transport artifacts were unlikely to be affecting the results (*SI Appendix*, Fig. S3 *A*–*D*). Given that all ZAP70 binding parameters were identified, we were able to use these data to estimate that CD45 has a high catalytic efficiency of 0.103 ± 0.01 µM^−1^ s^−1^ for ITAM3 (*SI Appendix*, Fig. S3*E*).

Although ZAP70 bound with the same affinity and kinetics to the ITAM3 peptide used in the previous section and the full-length Avi-CD3*ζ* ITAM3 used here (*SI Appendix*, Fig. S4), there seemed to be steric limitations to regulated unbinding from the shorter ITAM3 peptide since the CD45-mediated observed off-rate hit a plateau at the ZAP70 off-rate (*SI Appendix*, Fig. S5). Since there are only five amino acids between the biotin and first phosphotyrosine in the ITAM3 peptide, a likely explanation is that there is not enough space to accommodate both ZAP70 and CD45 near the streptavidin anchor. Consistent with this, we have previously observed strong steric hindrance of phosphatase domains accessing phosphotyrosines immobilized on SPR chip surfaces with short linkers (37).

### The Membrane Half-Life of ZAP70 in T Cells Correlates with the TCR–pMHC Half-Life.

The observation that dephosphorylation can accelerate the dissociation of ZAP70 from ITAMs suggests that the intracellular ZAP70–TCR half-life in T cells will not be fixed but will be dependent on local phosphatase activity. Interactions between TCR and pMHC are thought to occur in small (100- to 200-nm) close-contact regions of the T cell–antigen presenting cell interface that are depleted of CD45 (38–40). We hypothesized that if pMHC unbinding allows phosphorylated and ZAP70-bound TCR to diffuse out of regions depleted of CD45 and into regions with higher levels of CD45 (31), this would accelerate the unbinding of ZAP70, coupling the half-life of ZAP70 at the membrane to the half-life of the TCR–pMHC interaction. On the other hand, if regulated unbinding cannot operate, then the half-life of ZAP70 would be fixed and independent of the half-life of TCR–pMHC interactions. In both models, the number of ZAP70 molecules at the membrane would be expected to correlate with the TCR–pMHC half-life.

To test this hypothesis, we first established conditions for accurate off-rate measurements using imaging. We immobilized ITAM3 peptides on glass coverslips and used total internal reflection fluorescence microscopy together with single-particle tracking (SPT) to measure the binding time of ZAP70 tSH2-GFP or ZAP70 tSH2-Halotag-Alexa647. By fitting the distribution of ZAP70 binding times to exponentials, we were able to determine the off-rate for monophosphorylated ITAM3 N (11.4 ± 0.011 s^−1^) and ITAM C (4.75 ± 0.14 s^−1^) and biphosphorylated ITAM NC (0.368 ± 0.011 s^−1^) peptides (*Materials and Methods* and *SI Appendix*, Fig. S6). These kinetic rate constants were in agreement with those determined by SPR, validating the imaging conditions.

**Fig. 5.**
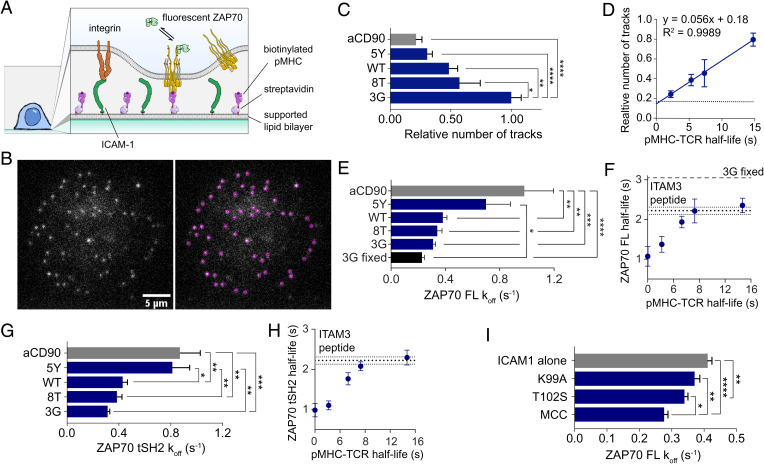
ZAP70 membrane half-life correlates with the TCR–pMHC half-life in T cells. (*A*) Diagram of the experimental system. (*B*) Example frame (*Left*) and identified particles (*Right*) of a live cell ZAP70-Halotag SPT experiment with 3G pMHC and ICAM1. (*C*) Number of labeled ZAP70s recruited to the interface between ILA Jurkats and pMHC-decorated supported lipid bilayer normalized to the highest-affinity pMHC (3G). (*D*) Number of ZAP70-Halotags over the TCR–pMHC half-life [measured at 25 ${}^{{}^{\circ}}$C (68)], with the horizontal line indicating the anti-CD90 condition. (*E*) Fitted *k*_off _and (*F*) half-life calculated from *k*_off _over the TCR–pMHC half-life from the distribution of membrane binding times. (*G* and *H*) Repeat of experiments in *E* and *F* except with the truncated tSH2-GFP instead of the full-length ZAP70-Halotag. (*I*) The *k*_off _of full-length ZAP70-GFP recruited to the membrane at the interface of primary and TCR transgenic mouse CD4${}^{+}$ T cells in live cell SPT experiments. All binding time distributions were fit with a sum of two exponentials with the slow rate displayed. Data are from at least eight cells per condition imaged in three separate experiments. Means ± SEMs are shown. *P < 0.05 (one-way ANOVA with Tukey’s posttest); **P < 0.01 (one-way ANOVA with Tukey’s posttest); ***P < 0.001 (one-way ANOVA with Tukey’s posttest); ****P < 0.0001 (one-way ANOVA with Tukey’s posttest). FL, full length; MCC, moth cytochrome c; WT, wild-type.

We next used supported lipid bilayers with biotinylated pMHC and ICAM1 to stimulate T cells and monitored the recruitment of fluorescent ZAP70 constructs (Fig. 5*A*). Full-length ZAP70-Halotag was introduced into ILA TCR-expressing Jurkat cells with normal endogenous expression of ZAP70 and labeled with low levels of Alexa647-Haloligand so that single molecules of ZAP70 recruited to the membrane could be monitored with SPT (Fig. 5*B*). As expected, the amount of membrane-recruited ZAP70 showed a strong correlation with the TCR–pMHC half-life (Fig. 5 *C* and *D*). Notably when pMHC was substituted for anti-CD90 in the bilayer to promote adhesion without TCR engagement, we still observed some residual recruitment of ZAP70 (Fig. 5*C*), consistent with the observation of ligand-independent TCR triggering (41–43).

In support of our hypothesis, measurements of the ZAP70 membrane half-life also correlated with the TCR–pMHC half-life (Fig. 5 *E* and *F*). As the TCR–pMHC half-live increased, we observed that the membrane half-life of ZAP70 appeared to reach a plateau at the rate we measured with isolated peptide and purified proteins (0.310 s^−1^ for 3G in Fig. 5 *E* and *F* compared with 0.368 s^−1^ in *SI Appendix*, Fig. S6*D*). The plateau was not a technical limit because trapping ZAP70 at the membrane by stimulating T cells with 3G and fixing prior to imaging showed a slower apparent off-rate (i.e., bleaching rate, 3G fixed in Fig. 5 *E* and *F*). Although we detected recruitment of ZAP70 without TCR engagement, it dissociated more rapidly (Fig. 5*E*, anti-CD90).

Recently, it has been suggested that Lck-mediated phosphorylation of interdomain B tyrosines in ZAP70 increases the half-life at the TCR (19). To control for this nonexclusive mechanism, which would also theoretically be regulated by phosphatases, we used the same tSH2-GFP construct used in SPR experiments. Despite lacking interdomain B, tSH2-GFP completely reproduced the results from full-length ZAP70-Halotag protein (Fig. 5 *G* and *H*), suggesting that phosphatase-regulated tandem SH2 binding to TCR ITAMs is sufficient to explain how the ZAP70 half-life is responsive to the TCR–pMHC half-life.

We also reproduced the results in primary mouse CD4${}^{+}$ T cells transgenic for the AND TCR, which recognizes a peptide generated from moth cytochrome C in the context of I-E*^k^*, presented with a set of altered peptide ligands. Although the TCR–pMHC kinetics are not known, functional data demonstrate that the index moth cytochrome c (MCC) peptide more robustly activates T cells compared with the T102S and K99A altered peptide ligands (44). In this system, we again observed recruitment of ZAP70 without engagement of TCR, this time with only ICAM1 in the bilayer (Fig. 5*I*). In agreement with the Jurkat results, we found significant differences in the ZAP70 membrane off-rate (Fig. 5*I*), supporting the hypothesis that the intracellular ZAP70–ITAM half-life is regulated and sensitive to the extracellular TCR–pMHC half-life.

Previously, it has been reported that single SH2 domains can remain at the membrane by rebinding to different phosphorylated tyrosines and extending the observed half-life by up to 20-fold (45), raising the possibility that differences in ZAP70 half-life are a result of rebinding rather than regulation by phosphatases. To address this, we applied the analysis used by Oh et al. (45) to our data and found no evidence for ZAP70 rebinding (*SI Appendix*, Fig. S8); the membrane half-life did not exceed the SPR half-life (*SI Appendix*, Fig. S8*A*), and the membrane diffusion coefficient of ZAP70, for pMHCs with different ZAP70 half-lives, did not correlate with its half-life (*SI Appendix*, Fig. S8 *B* and *C*). The rebinding mechanism was shown to require a high density of free binding sites (∼1,000 sites per 1 µm^2^) distributed on the micrometer scale where SH2 domains rebound across different receptors (45). This is unlikely to be the case for T cells where low pMHC densities generate spatially restricted TCR clusters. Additionally, we used stochastic spatial simulations and found that rebinding between the same ZAP70 and the same TCR is unlikely even if the TCR maintains 100 free ITAMs (*SI Appendix*, Fig. S7). This result is consistent with previous studies showing that rebinding to the same spatially localized site is not significant for cytosolic diffusion coefficients of D∼10 µm^2^/s, although it can be appreciable for typical membrane diffusion coefficients of D∼0.05 µm^2^/s (46–48). This result also agrees with the SPT experiments of Schwartz et al. (49), who found that Syk transiently interacted with Fc*ϵ*R1 clusters with dissociation kinetics that were independent of cluster size.

Lastly, the operational model of kinetic proofreading we initially introduced assumed the existence of a regulated unbinding rate that effectively coupled the half-life of ZAP70 to the TCR–pMHC half-life (Fig. 1*B*). Given that we had fully parametrized the rates of ZAP70–ITAM interactions (Fig. 3), we implemented a highly detailed molecular model of kinetic proofreading that included the detailed model of ZAP70 binding (*SI Appendix*, Fig. S9*A*). The model allows for ITAM and ZAP70 phosphorylation when the TCR is bound and dephosphorylation otherwise. In this molecular model, regulated unbinding is a consequence rather than an assumption of the model. To understand the contribution of regulated unbinding in this model, we also performed simulations where ZAP70 binding to ITAMs was based on the standard model where ZAP70 stably binds to ITAMs in a single dominant state. As expected, we found the highest levels of discrimination with high phosphatase activity and when using the detailed model of ZAP70 that allowed for dynamic internal states (*SI Appendix*, Fig. S9*B*). These improvements were a direct result of allowing TCRs to more rapidly reset after TCR–pMHC unbinding, thus preventing accumulation of ZAP70 on unbound TCRs and the consequent bypassing of proofreading. In these simulations, we noted that the amount of activated (i.e., phosphorylated) ZAP70 decreased more rapidly than the total amount of ZAP70 recruited as the TCR–pMHC off-rate increased. This is consistent with ZAP70 recruitment being an earlier step in the proofreading chain and therefore, being less sensitive to the TCR–pMHC off-rate.

## Discussion

Tandem SH2 domains were previously shown to increase affinity and specificity and to contribute to the allosteric activation of the kinase (10). Here, we report an additional feature: the ability to exhibit regulated unbinding to ITAMs.

The mechanism of regulated unbinding for ZAP70 is likely to be influenced by flexibility in the ITAM sequence. In ZAP70, the tandem SH2 domains are connected by a short coiled-coil region, and the N-terminal SH2 binding site includes some residues from the interface with the C-terminal SH2 domain (15, 50). These structural observations have suggested that the SH2 domains are locked together with little flexibility upon ITAM binding (10), but the disordered ITAM is likely to remain flexible so that individual phosphotyrosines can move away from either SH2 domain upon unbinding, allowing for phosphatase access. Thus, a highly kinetic binding mode can take place even if ZAP70 is relatively rigid. Although it is energetically less favorable, we note that ZAP70 may bind across phosphotyrosines from different ITAMs, as has been studied for Syk (35, 51). This was not included in our model and would be unlikely in our SPR experiments because we used a low density of immobilized ITAMs. Importantly, this binding mode would still allow regulated unbinding and may enhance it because of reduced steric hindrance for phosphatases. Our in vivo observation of regulated unbinding at the TCR demonstrates that even if cross-ITAM ZAP70 binding occurs, it does not hinder the regulation of the ZAP70 half-life. Whether this binding mode makes regulated unbinding more efficient is an interesting open question.

The efficiency of accelerating the dissociation of ZAP70 is dependent on the concentration of CD45. We have shown that the catalytic domain of CD45 can accelerate ZAP70 unbinding at concentrations larger than 10 µM using an in vitro SPR-based assay. In T cells, the CD45 catalytic domain and the TCR ITAMs are both tethered to the plasma membrane, and therefore, their physiological concentrations are difficult to determine. The volume they can explore proximal to the inner leaflet of the membrane is highly restricted, which increases their effective concentration. Estimates for other tethered phosphatase reactions suggest that effective concentrations as high as 1,000 µM are possible (37, 52). Therefore, the accelerated unbinding of ZAP70 we have observed in T cells may be a consequence of the combination of CD45 having a high abundance and high catalytic rate and being tethered near its substrates on the membrane. This supports the notion that the TCR can rapidly reverse its signaling state, including the dissociation of ZAP70 in T cells.

It is noteworthy that previous in vitro measurements of the ZAP70 half-life are significantly longer than those reported here. In early SPR measurements (12, 32), the tSH2 of ZAP70 did not unbind on the timescale of 100 s, suggesting that $k_{\text{off}}<0.01$ s^−1^, and this may be a consequence of the lower temperatures used in those experiments where affinities are known to be higher. A more recent study (33) using biolayer interferometry (BLI) reported $k_{\text{off}}∼10^{-4}$ s^−1^ (half-life of 1.9 h) for this interaction. Given that BLI is an optical technique that requires large amounts of protein binding for detection, the high amount of peptide immobilization used to achieve this may mean that a large amount of intermolecular rebinding across different ITAMs is taking place, leading to apparent long half-lives. Consistent with the present work, previous in vivo measurements of the ZAP70 half-life using fluorescence recovery after photobleaching have provided recovery timescales of ∼10 s (19, 53, 54), and measured half-lives of Syk for ITAMs agree well with our measurements [1.61 s for Syk binding Fc*ϵ*R1 in cells (49) and 0.52 s for binding an ITAM in vitro (36)].

Regulated unbinding is consistent with established mechanisms of ZAP70 activation and enhances our understanding of its functional role. Detailed analyses have shown that ZAP70 recruitment is accompanied by alignment of the SH2 domains that release ZAP70 from autoinhibition, allowing for phosphorylation that increases its activity (17, 19, 50, 55). As discussed above, it is likely that the cycling of ZAP70 between states that exposes ITAM tyrosines takes place while the SH2 domains remain aligned, allowing for ZAP70 phosphorylation. Given that ZAP70 recruitment is an earlier step in the kinetic proofreading chain, it follows that recruitment will be less sensitive to the TCR–pMHC half-life compared with ZAP70 phosphorylation or downstream signaling, and this is observed in our molecular model (*SI Appendix*, Fig. S9) and in experiments using a chimeric antigen receptor (CAR) system (56), respectively. This may explain the observation that ZAP70 recruitment to the TCR (but not activation) can readily be observed in the absence of high-affinity pMHCs (57, 58). Finally, it has been proposed that phosphorylation of Syk and ZAP70 in interdomain A can decrease their binding half-life (59, 60). In T cells, Katz et al. (60) have proposed that this leads to a “catch and release” mechanism for ZAP70 activation, whereby phosphorylation induces its unbinding from the TCR but sustains its signaling at the membrane. In our live cell SPT experiments, we observe that the half-life of membrane-recruited ZAP70 plateaus at the value we measured in SPR for ZAP70 on a phosphorylated ITAM (Fig. 5*F*). This suggests that phosphorylation of interdomain A may happen on a timescale that is similar to the natural off-rate of ZAP70 from the TCR, and therefore, the effect of phosphorylation on binding half-life of most recruitment events is minimal. However, interdomain A phosphorylation may be important for shortening the rare (as our modeling in *SI Appendix*, Fig. S7 suggests) rebinding events of active ZAP70 to phosphorylated TCRs, thus reducing sustained signaling and maintaining kinetic proofreading. Released, active ZAP70 may also be important since it could diffuse in the cytosol and phosphorylate linker for the activation of T cells (LAT) at distant membrane sites and nearby vesicles, which play a role in later stages of T cell activation (61, 62). A recent report suggested that the slower recovery of ZAP70 after photobleaching of CAR clusters was due to inefficient phosphorylation and subsequent release via the catch and release mechanism (54). Alternatively, this observation can be explained by a slower on-rate of ZAP70 binding to CARs, possibly because of the network of TNR receptor associated factors and signaling molecules recruited by the 41BB domain that may sterically hinder ZAP70 recruitment.

Finally, we note that regulated unbinding may be pervasive. There are nine other proteins with tandem SH2 domains and a large number of proteins with domains that bind regulated sites (i.e., a site that can be either on or off), which include SH2, phosphotyrosine-binding, forkhead-associated, and Pleckstrin homology (PH) domains. The qualitative feature of regulated unbinding cannot take place with one domain, and it does not require more than two. Interestingly, of the 447 proteins that contain these domains, there are no proteins that contain more than two domains per protein, with the exception of three PH domain–containing proteins (*SI Appendix*, Fig. S10). In contrast, constitutively binding domains, such as SH3, PDZ, C2, and WW domains, are found in copy numbers that exceed two on 66 proteins (*SI Appendix*, Fig. S10). In addition to acting between two proteins, regulated unbinding is likely to operate on stable phase-separated signaling assemblies formed by weakly binding multidomain proteins (63, 64). Interestingly, activated ZAP70 catalyzes the formation of LAT signaling assemblies (65), and recently, it has been suggested that LAT may be a proofreading step (66, 67). Consistent with this, in vitro–generated LAT assemblies displayed long half-lives without phosphatases but were disassembled within minutes in their presence (65), suggesting that multiple proteins within the TCR signaling cascade may exhibit regulated unbinding.

## Materials and Methods

A list of all materials used in the study, including plasmids and peptides used for measuring affinity and kinetics by SPR and those used in producing purified pMHC, can be found in *SI Appendix*. The protocols used for producing proteins (e.g., ZAP70 tSH2, CD45 catalytic domain, etc.) and the production and activation of T cells (e.g., Jurkat and primary murine T cells) can be found in *SI Appendix*. Detailed protocols for measuring the ZAP70 tSH2 affinity and kinetics by SPR and the membrane recruitment and lifetime of ZAP70 tSH2 and FL by total internal microscopy along with the analysis methods are provided in *SI Appendix*. Lastly, details on the three mathematical models used in the manuscript, including the operational model of kinetic proofreading (Fig. 1), the molecular model of kinetic proofreading (*SI Appendix*, Fig. S9), and the bivalent model of ZAP70 binding (Figs. 2 and 3), can be found in *SI Appendix*.

## Supplementary Material

Supplementary File

## Data Availability

All study data are included in the article and/or *SI Appendix*.
