# Supplementary material for: Dephosphorylation accelerates the dissociation of ZAP70 from the T cell receptor
Source: Proc Natl Acad Sci U S A. 2022 Feb 23;119(9):e2116815119. doi: 10.1073/pnas.2116815119 (PMC8892339; doi:10.1073/pnas.2116815119)
Supplement: Supplementary File [file pnas.2116815119.sapp.pdf]

Supplementary Information Appendix
Dephosphorylation accelerates the dissociation of ZAP70 from the T cell receptor

Jesse Goyette[1,2¶], David Depoil[3*], Zhengmin Yang[1], Samuel A. Isaacson[4], Jun Allard[5], P. Anton van der Merwe[6], Katharina Gaus[1,2,†], Michael L Dustin[3¶], Omer Dushek[6¶]

[1]EMBL Australia Node in Single Molecule Science, School of Medical Sciences, University of New South Wales, Sydney 2052, Australia.
[2]ARC Centre of Excellence in Advanced Molecular Imaging, University of New South Wales, Sydney 2052, Australia.
[3]The Kennedy Institute of Rheumatology, University of Oxford, OX3 7FY, Oxford, UK.
[4]Boston University, Department of Mathematics and Statistics, Boston, MA 02215.
[5]Center for Complex Biological Systems, University of California - Irvine, Irvine, CA.
[6]Sir William Dunn School of Pathology, University of Oxford, OX1 3RE, Oxford, UK.
[*]Present address: Evotec (UK) Ltd, 114 Innovation Drive, Milton Park, Abingdon, OX14 4RZ, UK
[†]Deceased.
[¶]Corresponding authors.

This file contains:

- SI Methods (Pages 2-11)
- SI Figures (Pages 12-24)

Supplementary Information Methods

Plasmids and peptides

For bacterial expression, a construct of the tandem SH2 domains of human ZAP70 (amino acids 1-264) and a C-terminal GFP or Halotag was cloned into pET21 between the NdeI and NotI restriction sites. For SPR experiments the cytoplasmic domain of CD3ζ (amino acids 52-164) with an N-terminal Avitag and a C-terminal hexahistadine tag was constructed by cloning the an Avitag-CD3ζ fusion into pET21 between the NdeI and NotI sites. For viral transduction full length human ZAP70 fused to Halotag were cloned into pHR-SIN-BX between the BamHI and NotI sites, removing the IRES-Emerald sequence. For *in vitro* transcription full length mouse ZAP70-GFP fusion was cloned into pGEM64 between AgeI and NotI.

All peptides were ordered from PeptideSynthetics and were certified to be >95% pure. Sequences of peptides used were ITAM3 N (biotin-GKGHDLLY*QGLSTATKDTYDALHMQ), ITAM3 C (biotin-GKGHDLLYQGLSTATKDTY*DALHMQ) and ITAM3 NC (biotin-GKGHDLLY*QGLSTATKDTY*DALHMQ), where Y* denotes a phopshotyrosine.

Protein production

ZAP70 tSH2-GFP, ZAP70 tSH2-Halotag, CD45 catalytic domain DNA constructs were transformed into the BL21 (DE3) strain (NEB) Escherichia coli and plated on LB agar with ampicillin (100 mg/ml), and then grown overnight at 37°C. For the avitag-CD3ζ ITAM3 peptide DNA construct BirA-transformed BL21 (DE3) Escherichia coli (BPS Bioscience) were used. The next day, colonies were innoculated into a 10 ml LB selection medium (LB medium with 100 mg/ml ampicillin), grown overnight at 37°C, and then transferred to 1 liter of LB selection medium until the optical density at 600 nm was 0.6 to 0.8. The cells were then treated with isopropyl-1-thio-D-galactopyranoside (final concentration, 0.5 mM) and harvested by centrifugation after 20 hours of culture at 18°C.

Bacterial pellets were resuspended in tris-buffered saline (TBS; 20 mM tris(hydroxymethyl)aminomethane, 150 mM NaCl) with protease inhibitors (protease inhibitor cocktail; Sigma), and then lysed with three 30 s bursts of sonication interspersed with 60 s rest periods on ice. Lysates were clarified by centriguation at 15,000 rcf followed by filtration through a 0.22 μm filter. Clarified lysates were applied to Ni^{2+}-NTA resin, which was washed with 10 column volumes of 2x PBS (10 mM phosphate, 300 mM NaCl), followed by 10 column volumes of 2x PBS with 10 mM imidazole, before His-tagged protein was eluted with 10 ml of 100 mM imidazole (pH 7.5). Protein was concentrated to 1 ml using a Amicon Ultra-4 Ultracel 30 kDa spin concentrator (Merck Millipore) and the protein was separated on a HiPrep 16/60 Sephacryl S-200 HR column (GE Healthcare) equilibrated with TBS. Peaks corresponding to the protein of interest were pooled, glycerol was added to a final concentration of 10% (v/v), and protein was stored in aliquots at -80°C until the day of experiment.

Avitag-CD3ζ ITAM3 was further phosphorylated *in vitro* using purified Lck in kinase buffer (TBS with 2 mM Mg_2SO_4, 500 μM ATP and 1 mM DTT) for 1 hour at room temperature.

On the day of the experiment, aliquots of proteins were thawed and buffer exchanged into 20 mM

HEPES, 150 mM NaCl, 1 mM EDTA, 0.005% Tween 20, and 1 mM dithiothreitol using a Zeba 7kDa spin column (Thermo Fischer). Concentrations of proteins were measured using the optical density at 280 nm, or 490 nm for GFP tagged proteins, using a DeNovix DS-11 FX spectrophotometer (Denovix).

Surface Plasmon Resonance

Experiments were performed on a Biacore S200 instrument (GE Healthcare Life Sciences). All experiments were performed at 37°C using HEPES-buffered saline (HBS-EP, 10 mM HEPES (pH 7.4), 150 mM NaCl, 1 mM EDTA, and 0.005% Tween P20) and a flow rate of 50 μl/min.

CM5 sensor chips were coupled with streptavidin to near saturation (typically between 4000 and 7000 RU) using the amine coupling kit (GE Healthcare Life Sciences). After streptavidin was coupled, biotinylated peptides were injected to give the indicated concentrations in experimental flow cells. For kinetic and equilibrium binding experiments excess biotin-binding sites were blocked with biotin in HBS-EP. Reference flow cells were treated with buffer and then blocked with biotin. The chip surface was then conditioned with 5 injections of HBS-EP followed by the indicated concentrations of ZAP70 constructs. Reference-subtracted data are shown.

For CD45 assays flow cells were not blocked with biotin after Avitag-CD3ζ ITAM3 peptide immobilisation. To perform the phosphatase assay an ABA injection method was used with ZAP70 tSH2-GFP (500 nM) as the flanking solution for an injection of a mixture of ZAP70 (500 nM) and CD45 at various concentrations. The flanking solution was first injected over a surface of Avitag-CD3ζ ITAM3 for 20 seconds to allow ZAP70 binding to reach equilibrium, then the mixture of ZAP70 tSH2-GFP and CD45 was injected. After each cycle a 30 s injection of 1x PhosSTOP (Merck) phosphatase inhibitors was performed and fresh biotinylated Avitag-CD3ζ ITAM3 immobilised.

All data fitting was performed in Prism (GraphPad) using the one site specific model for fitting steady-state data to determine K_D or the one phase exponential decay to determine k_{off} (both models included two fitting parameters and no issues with ambiguous fits were reported by Prism).

Cells

Jurkat T cells (Clone E6.1, ATCC) were maintained in RPMI 1640 (Gibco) supplemented with 10% (vol/vol) FCS, 2 mM L-glutamine, and 1 mM penicillin and streptomycin (all from Invitrogen). Jurkat cells stably expressing the ILA TCR were used in this study. The ILA TCR recognizes residues 540-548 (ILAKFLHWL) of the human telomerase reverse-transcriptase protein, presented in the context of the human MHC class I HLA-A*0201 (HLA-A2). To activate the ILA expressing Jurkat cells, monomeric, biotinylated human HLA-A2 complexed with the ILA peptide and several altered peptides (3G, 8T, 5Y) were used.

ZAP70-tSH2 GFP protein was introduced into ILA Jurkat cells by directly electroporating the purified protein (NEON; Invitrogen). Full-length ZAP70-Halotag was transduced into Jurkat cells using a lentiviral system and were labelled with Halotag ligand-Alexa647 made in house by reacting Halotag Amine ligand (Promega) with excess Alexa647-succinimidyl ester (Thermo Fischer) and neutralising excess reactive dye with glycine. To label cells 1 μM of Halotag ligand-Alexa647 in complete culture media was incubated

with cells for 30 min at 37°C, then the cells were washed twice with 20 ml of PBS, incubated at 37°C in complete media for 30 min, washed once more in PBS before imaging.

Primary CD4+ T cells were isolated from spleens of AND TCR transgenic mice using magnetic bead sorting. Messenger RNA encoding mouse ZAP70-GFP was transcribed *in vitro* and introduced into cells by electroporation 16 hours before imaging.

All cells were imaged in phenol-free RPMI with 10% FCS and 10 mM HEPES pH 7.5.

Total internal reflection microscopy and particle tracking

Supported lipid bilayers were created and loaded with pMHC and His-tagged ICAM-1 as described elsewhere (71). Live cell image sequences were acquired on a total internal reflection fluorescence (TIRF) microscope (ELYRA, Zeiss) with an environmental chamber equilibrated to 37°C. Chamber-slides with pMHC-decorated bilayers were equilibrated in the microscope environmental chamber at least 10 minutes before cells were added to wells and imaging commenced as soon as cells landed on the coverslip. Cells were imaged within 10 min of addition to the bilayer-containing wells.

GFP imaging was performed with 0.7 mW of 488 nm light and Alexa647 was imaged with 0.5 mW of 647 nm light. A 100 oil-immersion objective (NA = 1.46) was used for all imaging. For each cell, 500 images were acquired with a cooled, electron-multiplying charge-coupled device camera (iXon DU-897; Andor) with an exposure time of 200 ms. For experiments with ZAP70 tSH2-GFP binding to monophosphorylated peptides 1.4 mW 488 nm light and an exposure time of 50 ms was used.

Trackmate software (72) was used to identify and track single molecules of ZAP70-Halotag-Alexa647 or ZAP70 tSH2-GFP. Spot intensities showed a single gausian distribution (Fig S13) indicating that single molecules were being tracked.

For peptide binding experiments a peptide surface was generated by coating a coverslip surface with poly-L-lysine-PEG-biotin (PLL(20)-g[3.5]- PEG(2)/PEG(3.4)- biotin(20%), SuSoS Surface Technology), saturating the biotin with 10 μg/ml streptavidin in PBS/BSA (PBS with 1% w/v bovine serum albumin) for 30 min at room temp and incubating with biotinylated peptide. The surfaces were then washed thoroughly in PBS and ZAP70 tSH2-GFP or ZAP70 tSH2-Halo-Alexa647 was added at 200 nM in PBS/BSA with 1 mM DTT (to keep spurious disulfide bonds from forming) and the coverslips imaged as above for live cells.

The brightness and photobleaching of individual GFP molecules were determined by coating wells of a chamber coverslip in poly-L-lysine-PEG-biotin/streptavidin as above and adding a increasingly dilute biotinylated Avitag-GFP protein (produced in E.coli). For Alexa647, wells of a chamber coverslip were coated in poly-L-lysine and an increasing dilution of Alexa647-succinimidyl ester (Thermo Fischer) added and left to react for 30 min at room temperature. Wells with dilutions of Avitag-GFP or Alexa647 resulting in sparse single molecules with single step-bleaching characteristics were imaged.

Analysis of particle tracking

Cumulative distributions of track survival probability vs time were fit with exponential decay models using Graphpad Prism software. One- and two-phase exponential fits were tested and an extra sum-of-squares F test was used to determine whether a two-phase decay was necessary. A single-phase decay was sufficient to fit experiments using monophosphorylated ITAM immobilised on glass coverslips resulting in unbinding rates that agreed with SPR experiments (Fig. S11). For data with immobilised biphosphorylated ITAM a one-phase decay did not fit well and two-phase decay was necessary. The slower phase component of this fit agreed well with SPR data. Similarly a one-phase decay did not fit data from live cell experiments well and a two-phase decay was used. The fast phase component of fits from bivalent peptide on glass and cellular data was similar (Fig. S12A,B) suggesting that it was a result of fluorophore photophysics due to fluorophore 'blinking'. To demonstrate this, and to ensure that we were not missing an important short-lived ZAP70 state in T cells, we fixed T cells after stimulations and performed SPT finding that again, a two phase decay was needed with the slow phase now even slower than the ZAP70 SPR value (Fig. 5F, since it is fixed to the TIRF plane and tracks are lost by bleaching) but with a fast phase that again was similar to those observed before (Fig. S12A).

Mathematical models

Operational model of kinetic proofreading (Fig. 1)

The system of ordinary-differential-equations (ODEs) describing the operational model of kinetic proofreading with regulated unbinding (Fig. 1B) is as follows,

$$
\begin{aligned}
\dot{L} &= -k_{\text{on}}^{\text{R}} L R + k_{\text{off}}^{\text{R}} (C_0 + C_1 + C_2 + C_3) \\
\dot{R} &= -k_{\text{on}}^{\text{R}} L R + k_{\text{off}}^{\text{R}} (C_0 + C_1) + (k_{\text{off}} + k_{\text{reg}}) R_2 \\
\dot{C_0} &= +k_{\text{on}}^{\text{R}} L R - (k_{\text{off}}^{\text{R}} + k_{\text{p}}) C_0 \\
\dot{C_1} &= +k_{\text{p}} C_0 - (k_{\text{on}}^{*} + k_{\text{off}}^{\text{R}}) C_1 + k_{\text{off}} (C_2 + C_3) \\
\dot{C_2} &= +k_{\text{on}}^{*} C_1 - (k_{\text{off}}^{\text{R}} + k_{\text{off}} + k_{\text{p}}) C_2 + k_{\text{on}}^{\text{R}} L R_2 \\
\dot{C_3} &= +k_{\text{p}} C_2 - (k_{\text{off}} + k_{\text{off}}^{\text{R}}) C_3 \\
\dot{R_2} &= +k_{\text{off}}^{\text{R}} (C_2 + C_3) - k_{\text{on}}^{\text{R}} L R_2 - (k_{\text{off}} + k_{\text{reg}}) R_2
\end{aligned}
$$

where all chemical states and reaction rates are depicted in Fig. 1B and parameter values summarised in Table 2. The initial conditions were $L(t = 0) = L_T$, $R(t = 0) = R_T$, and all other states 0. The model was integrated to steady state using the Matlab (Mathworks, MA) function *ode15s* for the indicated values of k_{off} and k_{reg}. In this operational model, we have assumed that ZAP70 is in excess and is not depleted and therefore, have modelled recruitment using the first-order rate k_{on}^{*}.

The discrimination power was defined as the log of the fold-change in the ligand concentrations required to produce a concentration of 0.01 of active receptor ([Ligand]*) over the log fold-change in the off-rates of each ligand (see formula in Fig. 1C). Sensitivity was defined as [Ligand]* for the higher affinity ligand ($k_{\text{off}}^{\text{R}} = 1\ \text{s}^{-1}$). To calculate discrimination and sensitivity using the standard kinetic proofreading model, we used the previously reported analytical solution (22, 73) with N=0, 1, 2, or 3 steps and all other parameters as indicated in Table 2.

Table 2: Parameters for operational model of kinetic proofreading (Fig. 1)

Parameter	Description	Value	Units
R_T	TCR concentration	100	molecules/μm^2
L_T	pMHC concentration	1000	molecules/μm^2
k_{on}^{R}	TCR/pMHC on-rate	0.01	μm^{2}s^{-1}
$k_{\text{off}}^{\text{R}}$	TCR/pMHC off-rate	$10^{-0.5}$ (agonist)<br>$10^{1.5}$ (non-agonist)	s^{-1}
k_{p}	Phosphorylation rate	1	s^{-1}
k_{on}^{*}	Effective first-order ZAP70 on-rate	1	s^{-1}
k_{off}	ZAP70 off-rate	variable	s^{-1}
k_{reg}	Regulated ZAP70 off-rate	variable	s^{-1}

Bivalent ZAP70/ITAM binding model (Fig. 2)

The bivalent interaction of ZAP70 (with tandem SH2 domains) binding to phosphorylated ITAMs (with two phosphorylated tyrosines) that can be dephosphorylated by a phosphatase (Fig. 2D) is modelled using the following ordinary-differential-equations (ODEs),

$$
\begin{aligned}
\dot{R_{1,1}} &= -k_{\text{on}}^{\text{N}}L_T R_{1,1} + k_{\text{off}}^{\text{N}}B_{1,1} - k_{\text{on}}^{\text{C}}L_T R_{1,1} + k_{\text{off}}^{\text{C}}C_{1,1} - 2k_{\text{cat}}^{*}P_T R_{1,1} \\
\dot{R_{1,0}} &= -k_{\text{on}}^{\text{N}}L_T R_{1,0} + k_{\text{off}}^{\text{N}}A_{1,0} + k_{\text{cat}}^{*}P_T R_{1,1} - k_{\text{cat}}^{*}P_T R_{1,0} \\
\dot{R_{0,1}} &= -k_{\text{on}}^{\text{C}}L_T R_{0,1} + k_{\text{off}}^{\text{C}}A_{0,1} + k_{\text{cat}}^{*}P_T R_{1,1} - k_{\text{cat}}^{*}P_T R_{0,1} \\
\dot{R_{0,0}} &= k_{\text{cat}}^{*}P_T R_{1,0} + k_{\text{cat}}^{*}P_T R_{0,1} \\
\dot{A_{1,0}} &= k_{\text{cat}}^{*}P_T B_{1,1} + k_{\text{on}}^{\text{N}}L_T R_{1,0} - k_{\text{off}}^{\text{N}}A_{1,0} \\
\dot{A_{0,1}} &= k_{\text{cat}}^{*}P_T C_{1,1} + k_{\text{on}}^{\text{C}}L_T R_{0,1} - k_{\text{off}}^{\text{C}}A_{0,1} \\
\dot{B_{1,1}} &= +k_{\text{on}}^{\text{N}}L_T R_{1,1} - k_{\text{off}}^{\text{N}}B_{1,1} - k_{\text{on}}^{\text{C}}\sigma B_{1,1} + k_{\text{off}}^{\text{C}}D_{1,1} - k_{\text{cat}}^{*}P_T B_{1,1} \\
\dot{C_{1,1}} &= k_{\text{on}}^{\text{C}}L_T R_{1,1} - k_{\text{off}}^{\text{C}}C_{1,1} - k_{\text{on}}^{\text{N}}\sigma C_{1,1} + k_{\text{off}}^{\text{N}}D_{1,1} - k_{\text{cat}}^{*}P_T C_{1,1} \\
\dot{D_{1,1}} &= +k_{\text{on}}^{\text{C}}\sigma B_{1,1} - k_{\text{off}}^{\text{C}}D_{1,1} + k_{\text{on}}^{\text{N}}\sigma C_{1,1} - k_{\text{off}}^{\text{N}}D_{1,1}
\end{aligned}
$$

where subscripts indicate which of the two tyrosines are phosphorylated (e.g. 1,0 and 1,1 indicate phosphorylation of ITAM N only or both ITAM N and C, respectively), R represents unbound ITAM states with the indicated phosphorylation, A represents bound ITAM states that are monophosphorylated, B and C are biphosphorylated ITAM states where only ITAM N or ITAM C are bound by a ZAP70 SH2 domain, respectively, and D is a biphosphorylated ITAM with both tyrosines bound to the tandem SH2 domains of ZAP70. The model parameters are described in the text and summarised in Table 3. The model was numerically solved in Matlab (Mathworks, MA) using *ode15s*.

The mathematical model makes several assumptions that we make explicit here. First, phosphatases can only dephosphorylate tyrosines that are not bound to SH2 domains, as previously described (34). Second, the absolute amount of phosphorylated tyrosines is small compared to the absolute amount of ZAP70 or phosphatases so that they are not depleted, which allows for these molecules to have a fixed concentration (L_T and P_T, respectively). This is a standard assumption when modelling SPR experiments where they are replenished by flow and is reasonable for cellular experiments because only a fraction of TCRs will be phosphorylated. Third, only a single molecule of ZAP70 can interact with an ITAM. This is reasonable

Table 3: Parameters for bivalent ZAP70/ITAM binding model

Parameter	Description	Units
L_T	Concentration of ZAP70	μM
k_{on}^{N}	ITAM N on-rate	$\mu\text{M}^{-1}\text{s}^{-1}$
$k_{\text{off}}^{\text{N}}$	ITAM N off-rate	s^{-1}
k_{on}^{C}	ITAM C on-rate	$\mu\text{M}^{-1}\text{s}^{-1}$
$k_{\text{off}}^{\text{C}}$	ITAM C off-rate	s^{-1}
σ	Local concentration	μM
P_T	Phosphatase concentration	μM
k_{cat}	Catalytic efficiency of phosphatase	$\mu\text{M}^{-1}\text{s}^{-1}$

because the maximum concentration of ZAP70 binding to ITAM3 NC (0.6 μM) is much lower than the experimentally observed σ (1161 μM) so that solution ZAP70 could not compete with ZAP70 already bound to the ITAM. Fourth, no cross reactions were included that would allow, for example, ITAM N to bind not only C-SH2 of ZAP70 but also the N-SH2. This is reasonable because we could not find any evidence for these reactions and this is underlined by the fact that the present simpler model was sufficient to explain the data. Fifth, ZAP70 binding is defined as all states where ZAP70 is interacting with an ITAM (i.e. $A_{1,0} + A_{0,1} + B_{1,1} + C_{1,1} + D_{1,1}$). This is reasonable because both in SPR and in microscopy, the microscopic states of ZAP70 are not resolved but rather total localisation is reported.

The calculations in Fig. 2 were initialised with all states 0 except $R_{1,1}$ which was set to 1. The concentration of ZAP70 remained fixed for the entire calculation ($L_T = 1$ μM) whereas the concentration of phosphatase (P_T) was initially 0 for 30 seconds to allow the system to come to steady-state before increasing to the indicated concentration with $k_{\text{cat}} = 0.1$ $\mu\text{M}^{-1}\text{s}^{-1}$. The bivalent calculations (Fig. 2D,E) used $k_{\text{on}}^{\text{N}} = k_{\text{on}}^{\text{C}} = 1$ $\mu\text{M}^{-1}\text{s}^{-1}$, $k_{\text{off}}^{\text{N}} = k_{\text{off}}^{\text{C}} = 10$ s^{-1}, and $\sigma = 1000$ μM resulting in an effective unbinding rate back to solution of 0.2 s^{-1}. The monovalent calculations (Fig. 2A,B) used the bivalent model with $k_{\text{on}}^{\text{N}} = 1$ $\mu\text{M}^{-1}\text{s}^{-1}$ and $k_{\text{off}}^{\text{N}} = 0.2$ s^{-1} but with $k_{\text{on}}^{\text{C}} = 0$ so that only a single SH2 domain can interact.

The SPR experiments provide the amount of ZAP70 bound at steady-state for different concentrations of ZAP70 on the biphosphorylated ITAM NC peptide. To calculate this in the mathematical model above, we set $P_T = 0$ (no phosphatase) and solved the ODE system in the steady-state to calculate total ZAP70 binding (Y, defined as $Y = A_{1,0} + A_{0,1} + B_{1,1} + C_{1,1} + D_{1,1}$),

$$Y = \frac{L_T R_T}{K_{\text{D}} + L_T} \tag{1}$$

where R_T is the total peptide concentration, L_T is the ZAP70 concentration, and K_{D} is the effective ZAP70 affinity ($K_{\text{D}} = K_{\text{D}}^{\text{N}} K_{\text{D}}^{\text{C}} / (\sigma + K_{\text{D}}^{\text{N}} + K_{\text{D}}^{\text{C}})$). This equation derived from the bivalent model has an identical form to that of the monomeric binding, which explains why an apparent monovalent model is able to fit the bivalent interaction of ZAP70.

Molecular model of kinetic proofreading (Fig. S9)

The rule-based framework of BioNetGen (74) was used to generate a molecular model of ZAP70 activation (see Supplementary Information for the full code). This framework is particularly suited for molecular models where the number of chemical states can become very large as a result of reactions that take place independently. The model included TCR/pMHC binding that initiated ITAM phosphorylation (2 sites), ZAP70 binding by the detailed model (using the experimentally determined parameters), and ZAP70 phosphorylatio on 4 sites. Kinetic proofreading was realised by allowing for phosphorylation (ITAM and ZAP70) only when the TCR is bound and dephosphorylation only when the TCR is unbound. Importantly, ZAP70 binding was dependent on ITAM phosphorylation but independent of TCR/pMHC binding. In this way, regulated unbinding of ZAP70 naturally arose out of the explicit modelling of microscopic binding ZAP70-ITAM binding states and phosphatase activity. These reactions produced a total of 64 distinct chemical states and 270 chemical reactions between these states (see Fig. S9 for a schematic of a subset of these states and reactions).

The model was numerically solved in Matlab (Mathworks, MA) using *ode15s* to the steady-state. The amount of TCR bound ZAP70 (either with one or two SH2 domains) and the amount of activated ZAP70 (defined as the amount of bound ZAP70 that is phosphorylated on all 4 sites) was calculated for different TCR/pMHC off-rates using different phosphatase activities at the membrane. The model parameters are summarised in Table S4. For the detailed model of ZAP70 binding the experimental parameters for each individual SH2 domain were used (Table 1) along with the estimate of the local concentration ($\sigma = 1161$ μM). For the standard ZAP70 binding model, kon2 in the model was set to zero with kon1 and koff1 set equal to the rates determined for ZAP70 binding ITAM NC (Table 1).

Table 4: Parameters for molecular model of kinetic proofreading

Parameter	Description	Value	Units
R	Number of TCRs	31000	-
L	Number of pMHCs	310000	-
Z	Number of ZAP70s	130000	-
k_{on}	TCR/pMHC on-rate	0.01	μm^2/s
k_{off}	TCR/pMHC off-rate	varied	s^{-1}
kpr	Phosphorylation rate of ITAM	1	s^{-1}
kpe	Phosphorylation rate of ZAP70	4	s^{-1}
km	Dephosphorylation rate at the membrane	varied	s^{-1}
kc	Dephosphorylation rate in cytosol	10^8	s^{-1}

Stochastic spatial simulations (Fig. S7)

To investigate the rebinding of a cytosolic protein (particle) to a surface receptor, we utilised two independent spatial stochastic simulation algorithms that produced identical results. The Brownian Dynamics algorithm (75) is a continuous-space discrete-time algorithm whereas the Gillespie algorithm is a discrete-space (on lattice) continuous-time algorithm (76, 77). The surface receptor was assumed to be fixed (immobile) at the origin ($r = 0$). The cytosolic protein was assumed to diffuse in the cytoplasm ($D = 10$ μm^2/s) with

diffusion is limited to the half-space below the membrane (i.e. reflecting boundary at the membrane). The particle can reversibly bind to the receptor with first-order kinetics (k^*_{on} and $k_{\text{off}} = 0.2$ s^{-1}), provided it is within a distance $b = 10$ nm of the origin, where b is the distance explored by the cytoplasmic tail of the receptor. The on-rate was proportional to the bimolecular on-rate, the number of binding sites, and the volume (proportional to b): $k^*_{\text{on}} = k_{\text{on}} \times N \times 1/V$, where $k_{\text{on}} = 1.89$ μM^{-1}s^{-1}, N is the number of ITAMs, and V is the reaction volume ($V = ((4/3)\pi b^3)/2$). The simulations were initialised with the cytosolic protein having just unbound from the receptor and therefore, it was placed randomly within the half-sphere determined by b. The simulations were terminated when the cytosolic protein diffused beyond $r = a$. We took $a = 150$ nm, which is approximately the mean distance between ZAP70 molecules within the cytoplasm. The simulations were repeated 10^6 times and the mean first passage time to reach a and the number of rebinding events within this time were recorded for different values of N.

BioNetGen BNGL file for molecular model of kinetic proofreading

```
#BNGL file for molecular model for kinetic proofreading

begin parameters

#TCR/pMHC binding (koff varied)
kon 0.01
koff 1

#ZAP70 Binding
kon1 0.26
koff1 5.5
kon2 1.52
koff2 11.4
sigma 1161

#ZAP70 Intramolecular reactions
kon1s   kon1*sigma
koff1s  koff1
kon2s   kon2*sigma
koff2s  koff2

#TCR Phosphorylation (when pMHC is bound to TCR)
kpr     1

#ZAP70 Phosphorylation (when pMHC and ZAP70 are bound to TCR)
kpe     4

#Dephosphorylation rate at the membrane (varied)
km      2500

#Dephosphorylation rate in solution
kc      1e8
```

```
end parameters

begin molecule types
    L(b)
    R(b,Y1~U~P,Y2~U~P)
    Z(b1,b2,Y~U~P~2P~3P~4P)
end  molecule types

begin seed species
    L(b)            310000
    R(b,Y1~U,Y2~U)  31000
    Z(b1,b2,Y~U)    130000
end seed species

begin observables
    Molecules BoundReceptor R(b!+)
    Molecules BoundEffector Z(b1!+,b2),Z(b1,b2!+),Z(b1!+,b2!+)
    Molecules BoundActiveEffector Z(b1!+,b2,Y~4P),Z(b1,b2!+,Y~4P),Z(b1!+,b2!+,Y~4P)
    Molecules ActiveEffector Z(Y~4P)
end observables

begin reaction rules

#TCR/pMHC binding
L(b) + R(b) <-> L(b!1).R(b!1) kon,koff

#TCR phosphorylation
R(b!+,Y1~U) -> R(b!+,Y1~P) kpr
R(b!+,Y2~U) -> R(b!+,Y2~P) kpr

#ZAP70 binding
R(Y1~P,Y2~U) + Z(b1,b2) <-> R(Y1~P!1,Y2~U).Z(b1!1,b2)   kon1,koff1
R(Y1~U,Y2~P) + Z(b1,b2) <-> R(Y1~U,Y2~P!1).Z(b1,b2!1)   kon2,koff2

R(Y1~P,Y2~P) + Z(b1,b2) <-> R(Y1~P!1,Y2~P).Z(b1!1,b2)   kon1,koff1
R(Y1~P,Y2~P) + Z(b1,b2) <-> R(Y1~P,Y2~P!1).Z(b1,b2!1)   kon2,koff2

R(Y1~P!1,Y2~P).Z(b1!1,b2) <-> R(Y1~P!1,Y2~P!2).Z(b1!1,b2!2) kon2s,koff2s
R(Y1~P,Y2~P!1).Z(b1,b2!1) <-> R(Y1~P!2,Y2~P!1).Z(b1!2,b2!1) kon1s,koff1s

#ZAP70 phosphorylation
Z(b1!+,b2,Y~U) -> Z(b1!+,b2,Y~P) kpe
Z(b1!+,b2,Y~P) -> Z(b1!+,b2,Y~2P) kpe
Z(b1!+,b2,Y~2P) -> Z(b1!+,b2,Y~3P) kpe
Z(b1!+,b2,Y~3P) -> Z(b1!+,b2,Y~4P) kpe

Z(b2!+,b1,Y~U) -> Z(b2!+,b1,Y~P) kpe
```

```
Z(b2!+,b1,Y~P) -> Z(b2!+,b1,Y~2P) kpe
Z(b2!+,b1,Y~2P) -> Z(b2!+,b1,Y~3P) kpe
Z(b2!+,b1,Y~3P) -> Z(b2!+,b1,Y~4P) kpe

Z(b2!+,b1!+,Y~U) -> Z(b2!+,b1!+,Y~P) kpe
Z(b2!+,b1!+,Y~P) -> Z(b2!+,b1!+,Y~2P) kpe
Z(b2!+,b1!+,Y~2P) -> Z(b2!+,b1!+,Y~3P) kpe
Z(b2!+,b1!+,Y~3P) -> Z(b2!+,b1!+,Y~4P) kpe

#Receptor dephosphorylation
R(b,Y1~P) -> R(b,Y1~U)  km
R(b,Y2~P) -> R(b,Y2~U)  km

#ZAP70 dephosphorylation (at membrane)
R(b).Z(Y~4P) -> R(b).Z(Y~3P) km
R(b).Z(Y~3P) -> R(b).Z(Y~2P) km
R(b).Z(Y~2P) -> R(b).Z(Y~P) km
R(b).Z(Y~P) -> R(b).Z(Y~U) km

#ZAP70 dephosphorylation (in solution)
Z(b1,b2,Y~4P) -> Z(b1,b2,Y~3P) kc
Z(b1,b2,Y~3P) -> Z(b1,b2,Y~2P) kc
Z(b1,b2,Y~2P) -> Z(b1,b2,Y~P) kc
Z(b1,b2,Y~P) -> Z(b1,b2,Y~U) kc

end reaction rules


begin actions

generate_network({overwrite=>1});
writeMfile({});

end actions
```

Supplementary Information Figures

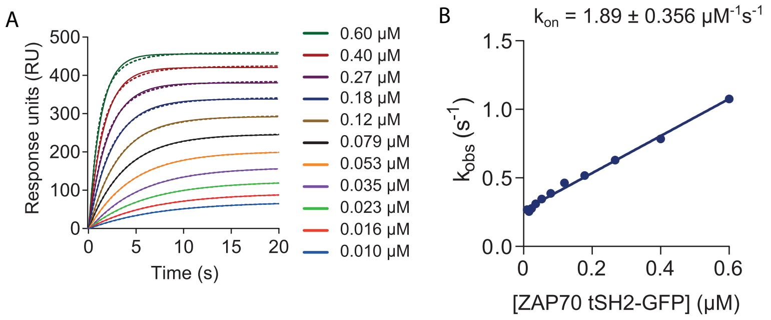

Figure S1: Observed on-rate (k_{obs}) analysis of tSH2-GFP interacting with biphosphorylated ITAM3. (A) Representative association phase data (dots) and exponential association phase fit (solid lines). (B) Fitted rate constant (k_{obs}) over [tSH2-GFP] with a linear fit ($k_{obs} = k_{on}[\text{tSH2-GFP}]+k_{off}$). The value of k_{on} (slope) is indicated. Related to Fig. 3

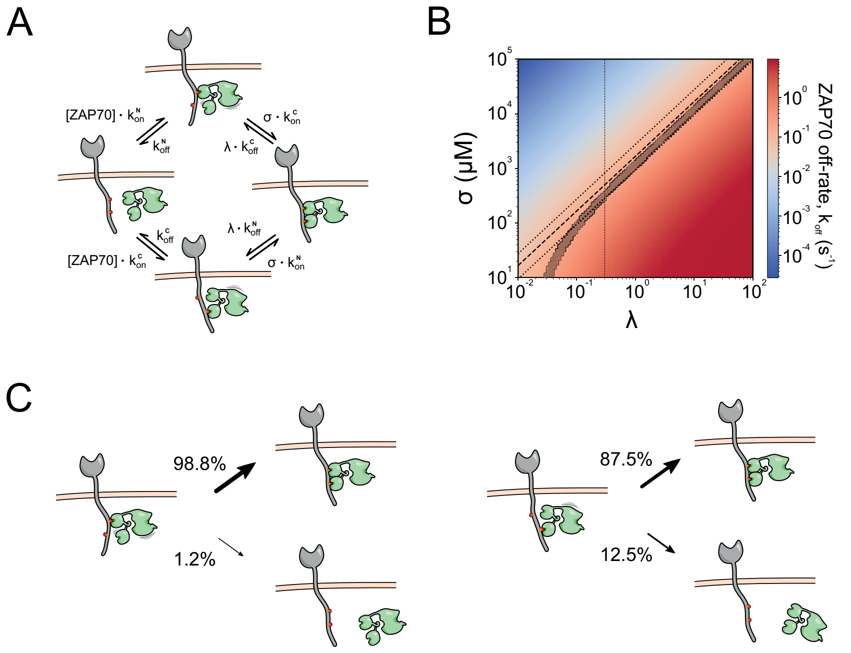

Figure S2: Model of ZAP70/ITAM interaction that includes conformational cooperativity predicts dynamic rebinding of SH2 domains. (A) The model of ZAP70/ITAM interaction (Fig. 2D) was modified to include a conformational cooperativity parameter (λ) that multiplied the off-rates only when ZAP70 is bound to both ITAM tyrosines. Since λ modifies the $k_{\text{off}}^{\text{N}}$ and $k_{\text{off}}^{\text{C}}$ rates, values of $\lambda < 1$ represent positive co-operativity, whereas $\lambda > 1$ represent negative co-operativity. (B) Heap map of ZAP70 off-rate predicted by the mathematical model when varying σ and λ. The 4 kinetic rate constants (2 for each SH2 domain) were fixed to their experimentally determined values (Table 1). Regions within the range of k_{off} ±SEM measured experimentally are shaded in grey. The dashed line on the diagonal is the experimentally determined ratio of σ/λ using the dissociation constants ($\sigma/\lambda = (K_{\text{D}}^{\text{N}} K_{\text{D}}^{\text{C}} - K_{\text{D}}(K_{\text{D}}^{\text{N}} + K_{\text{D}}^{\text{C}}))/K_{\text{D}}$) with the dotted lines indicating upper and lower bounds. The best overlap occurs for values of $\lambda > 0.3$ (indicated by a dashed vertical line). (C) Probability of rebinding or unbinding when ZAP70 is bound to N- (left panel) or C-terminal (right panel) tyrosines of ITAM3 if $\lambda = 0.3$ and $\sigma = 300$ μM.

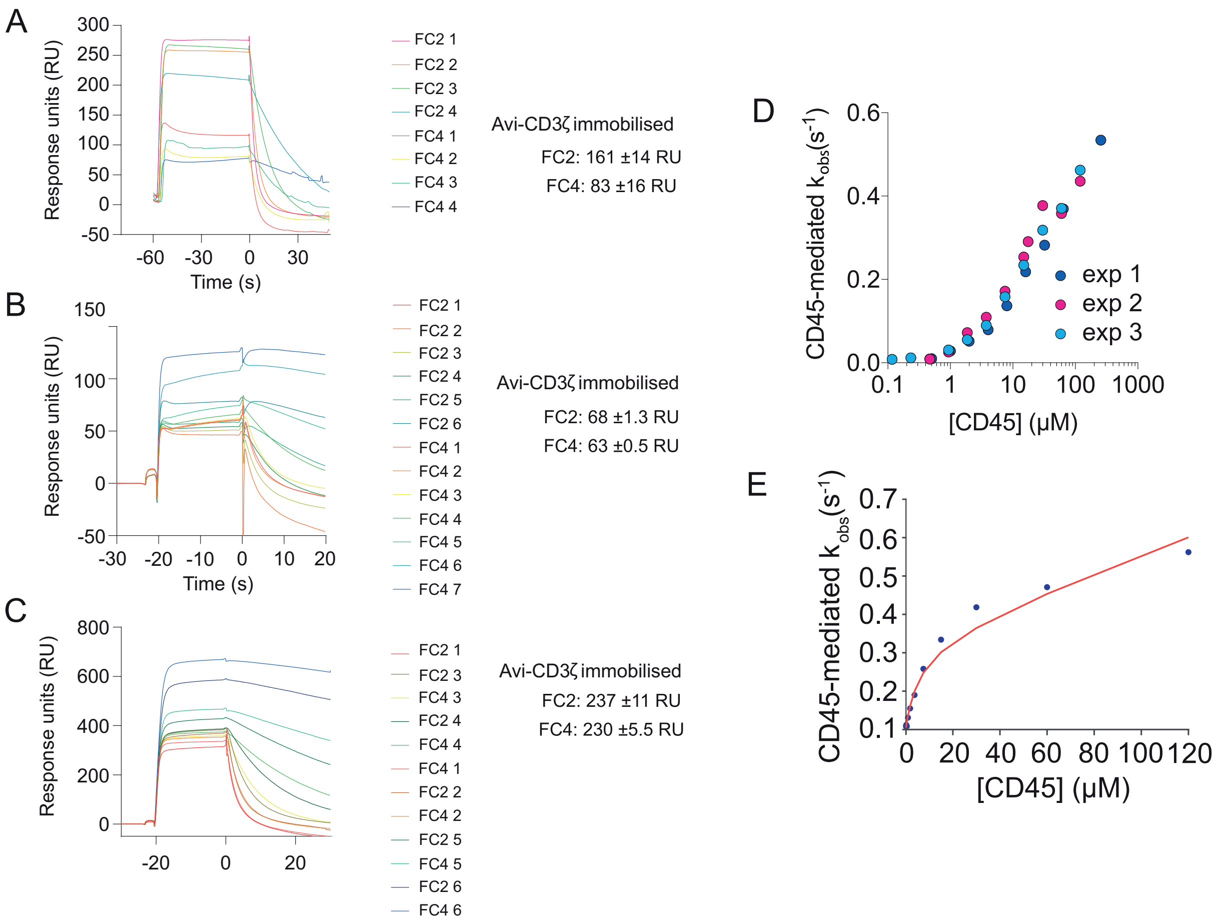

Figure S3: The CD45-mediated observed off-rate of ZAP70 is similar at different Avi-CD3ζ ITAM3 peptide immobilisation levels. (A-C) Overlay of sensograms from individual experiments differing in Avi-CD3ζ ITAM3 peptide immobilisation. The mean ±SD of peptide immobilised per cycle is shown to the right of the sensograms. (D) The fitted CD45-mediated observed off-rate over [CD45] for experimental data shown in panels A (exp 1), B (exp 2) and C (exp 3). (E) The detailed model of ZAP70-ITAM binding is fit to the CD45-mediated k_{obs} (red curve) with all binding kinetics fixed to their experimentally determined values with the catalytic efficiency of CD45 dephosphorylating CD3ζ ITAM3 ($k_{\text{cat}}/K_{\text{M}}$) being a fitted parameter. The mean and SEM across N=4 independent experiments produced a value of 0.103±0.01 μM^{-1} s^{-1}.

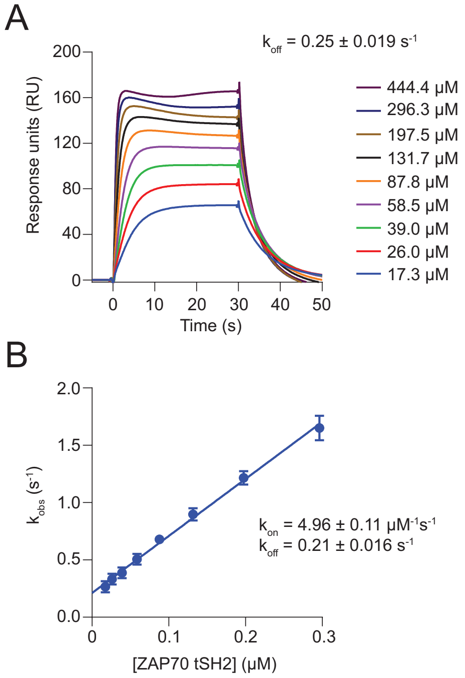

Figure S4: Binding kinetics of ZAP70 interacting with full length intracellular CD3ζ peptide phosphorylated on ITAM3. (A) Example sensograms from ZAP70 tSH2-GFP protein binding to phosphorylated Avi-CD3ζ. The dissociation rate (k_{off}) derived from fits of the dissociation phase of 4 experiments $\pm$SEM is shown. (B) Plot of k_{obs} vs concentration of tSH2-GFP, k_{on} and k_{off} $\pm$SE from the slope and intercept of the linear regression are shown.

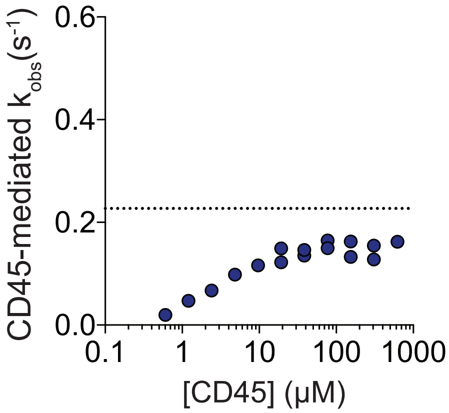

Figure S5: The CD45-mediated observed off-rate of ZAP70 tSH2-GFP measured on short biphosphorylated ITAM3 peptide over [CD45] plateaus near the value of k_{off} for ZAP70 tSH2-GFP for ITAM3 (shown as a horizontal dotted line).

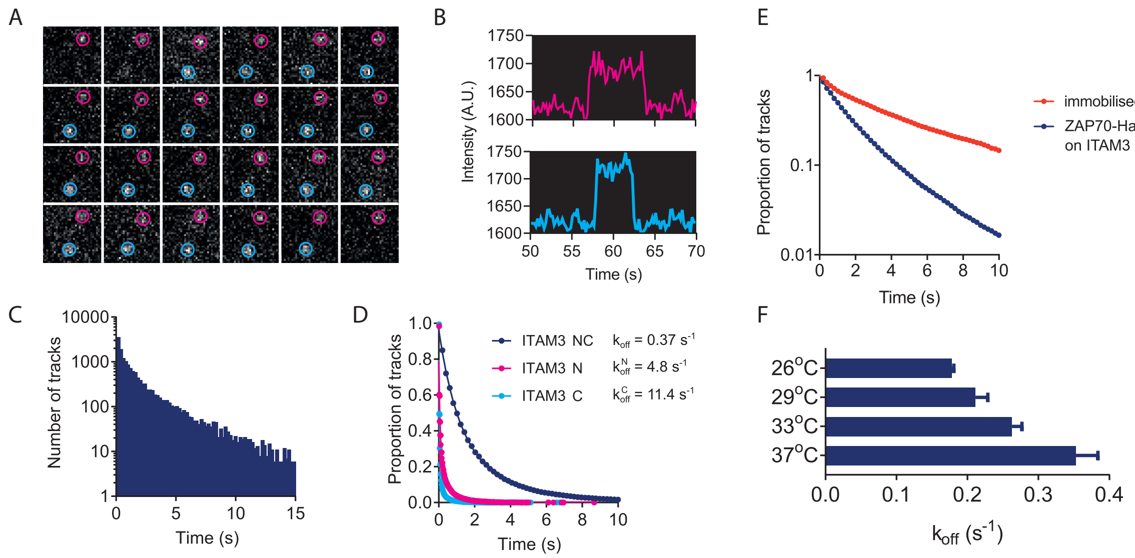

Figure S6: Unbinding kinetics measured by SPT using TIRF microscopy are similar to those measured by SPR. (A) Example 2.9 x 2.9 μm region from TIRF imaging showing single ZAP70 tSH2-GFP molecules (outlined in magenta and teal circles) binding to biphosphorylated CD3ζ ITAM3 peptide immobilised on a glass coverslip. Images were acquired at 5 Hz. (B) Intensity vs time trace of regions containing particles in panel A showing single step increases and decreases indicative of single molecule binding and unbinding events. (C) Example histogram of track lengths from SPT of ZAP70 tSH2-GFP binding to biphosphorylated CD3ζ ITAM3 peptide immobilised on a coverslip surface. Images were acquired at 5 Hz for 400 seconds. (D) Track lifetimes of tSH2-GFP binding to N-terminally phosphorylated, C-terminally phosphorylated and biphosphorylated CD3ζ ITAM3 peptide with eponential fit (line). Data are representative of 16 image stacks taken over 3 independent experiments. (E) Photobleaching minimally impacts on k_{off} measurements. Bleaching rate of Alexa647-PLL sparsely immobilised on glass (immobilised Alexa647) compared with the lifetime of ZAP70 tSH2-Halotag labelled with Alexa647-haloligand binding to immobilised biphosphorylated ITAM3 peptide (ZAP70-Halo-Alexa647 on ITAM3 NC). Both were imaged at 37°C using identical imaging conditions. (F) k_{off} of ZAP70 tSH2-GFP binding to biphosphorylated ITAM3 peptide at different temperatures demonstrating the ability to measure longer binding lifetimes.

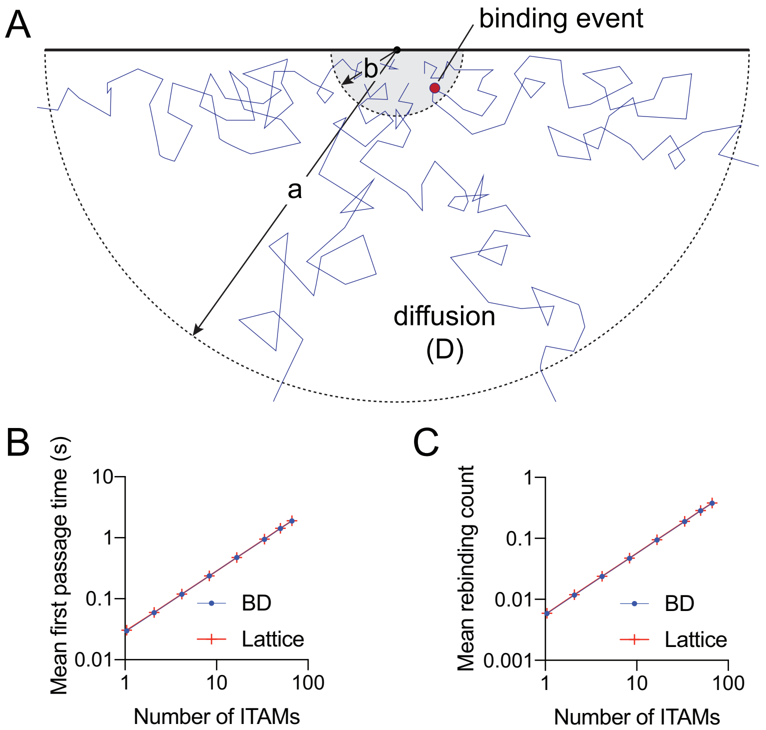

Figure S7: Stochastic spatial simulations reveal limited rebinding between the same cytosolic protein and the same surface receptor. A) Simulation schematic. The cytoplasmic tail of the surface receptor is assumed to explore the half-sphere of radius b and the cytosolic protein can rebind the receptor within this half-sphere with first-order kinetics. The simulation is initiated with the cytosolic protein unbound from the surface receptor and placed randomly within the binding half-sphere. The simulation is terminated when the cytosolic protein diffuses beyond the half-sphere of radius a. B) The average time to diffuse beyond the half-sphere of radius a (mean first passage time) over the number of binding sites (ITAMs). C) The average number of rebinding events over the number of binding sites. See Methods for simulation details.

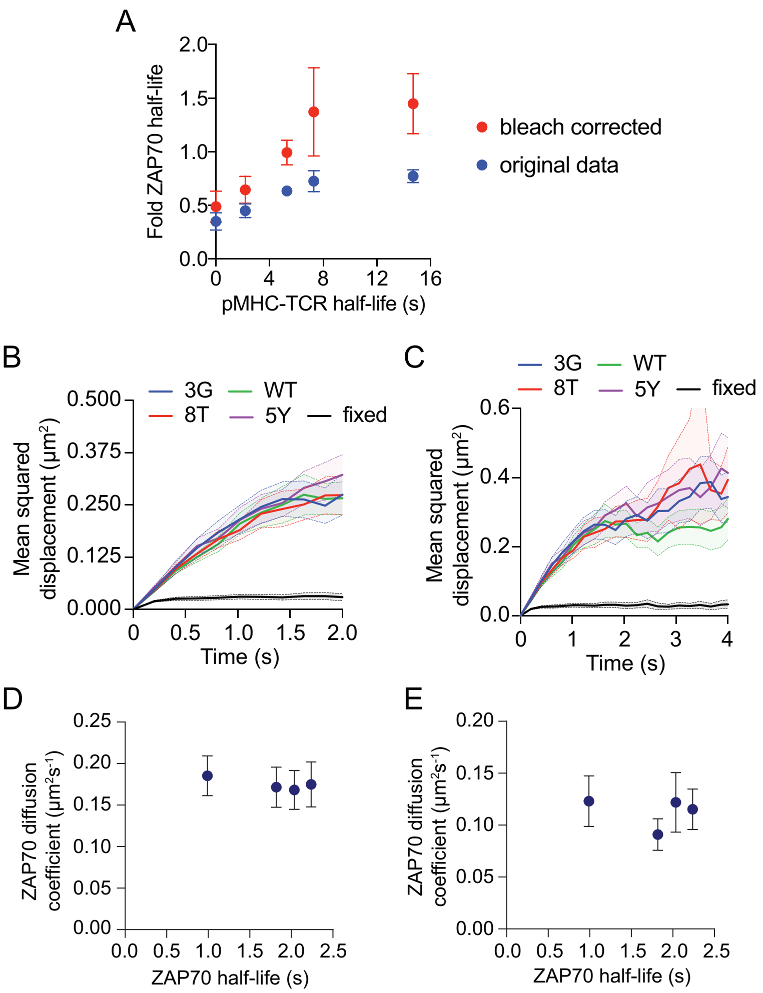

Figure S8: No evidence that the same ZAP70 protein rebinds the TCR. A) The fold-change in ZAP70 membrane over SPR half-life for all TCR/pMHC interactions tested. B-C) The mean squared displacement on short (panel B) and long (panel C) timescales for different pMHCs. D-E) Diffusion coefficients derived from the truncated short timescale data (panel D) and the complete data (panel E) over the ZAP70 membrane half-life .

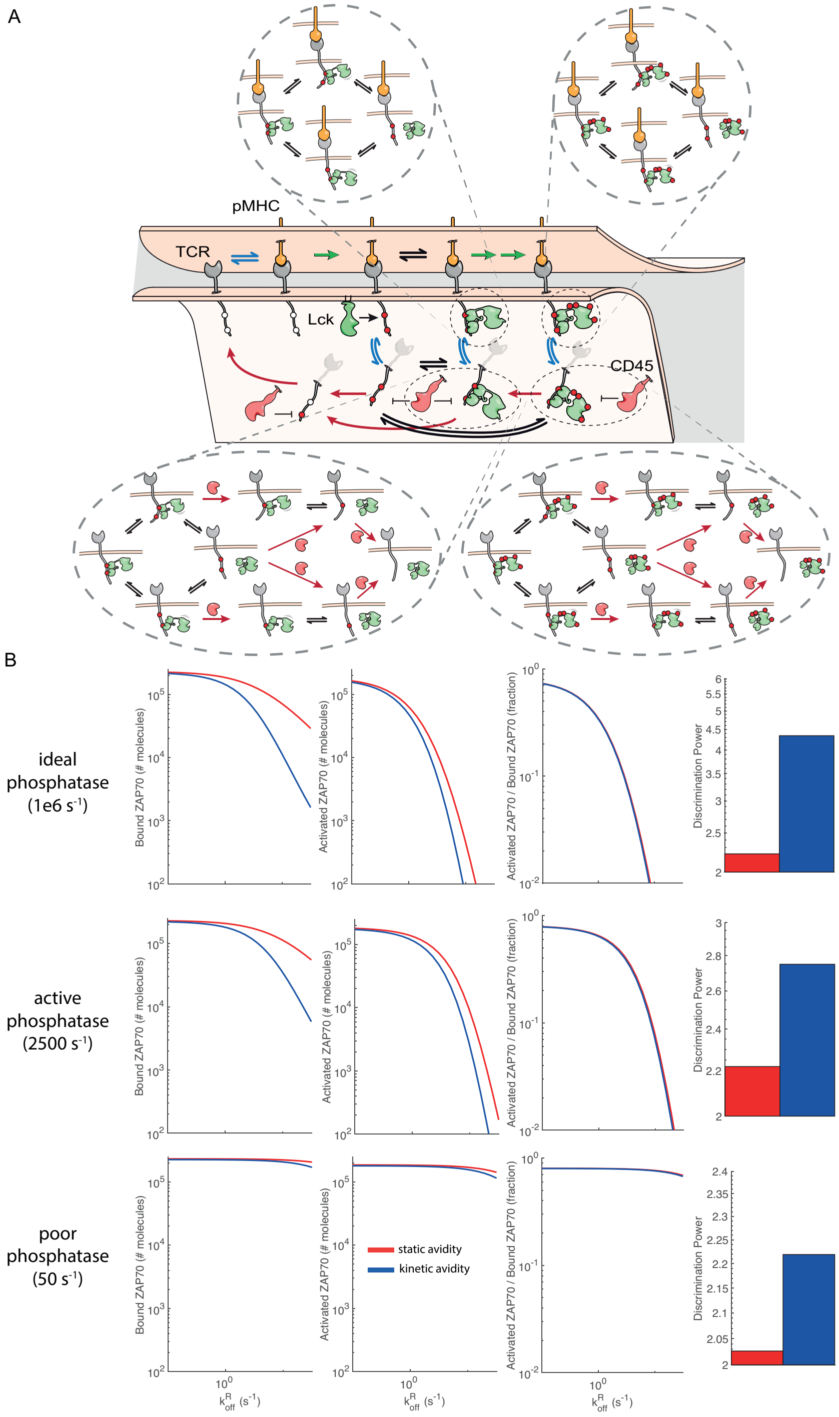

Figure S9: Molecular model of kinetic proofreading. A) Simplified schematic of the molecular proofreading model that includes TCR/pMHC binding (blue arrows), phosphorylation of the TCR and ZAP70 when the TCR is bound to pMHC (green arrows), dephosphorylation when the TCR is unbound from pMHC (red arrows), and ZAP70 binding (black arrows). Bound ZAP70 is defined by the amount of ZAP70 interacting with the TCR with either one or two SH2 domains and activated ZAP70 is defined as the amount of bound ZAP70 fully phosphorylated. B) The amount of ZAP70 bound (1st column), activated (2nd column), or the ratio of activated to bound (3rd column) over the TCR/pMHC off-rate (x-axis) when using an ideal (first row), an active (second row), or a poor (third row) phosphatase. The discrimination power (4th column, as defined in Fig. 1) for each case. See Methods for computational details.

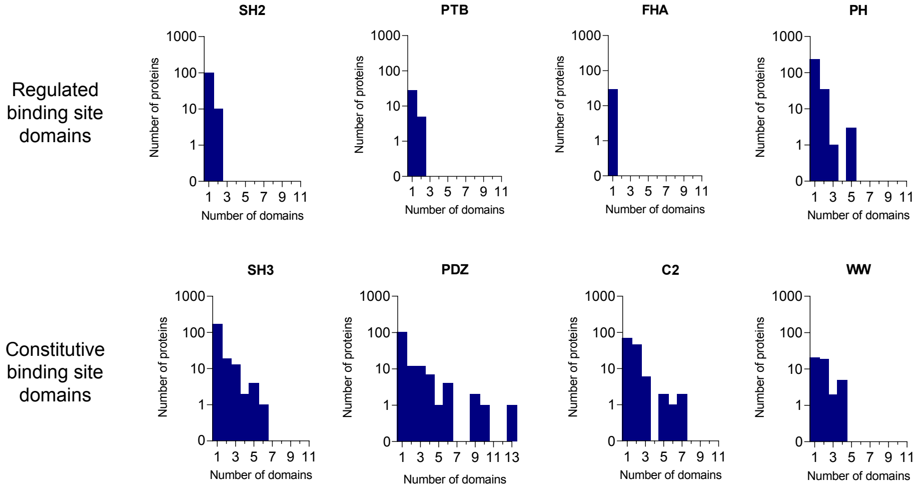

Figure S10: Numbers of genes in the human genome with single or multiple repeated binding domains. Data for Src homology 2 (SH2), Src homology 3 (SH3), C2, Pleckstrin homology (PH), phosphotyrosine binding (PTB), PDZ, forkhead-associated (FHA) and WW domains are shown. Shown are the number of proteins (y-axis) that have the indicated number of binding domains (x-axis) for each domain family. Note that SH2, PTB, FHA, and PH domains bind to regulated sites that can be either 'on' or 'off' (e.g. SH2 and PTB domains bind to phosphorylated but not dephosphorylated tyrosines) whereas SH3, PDZ, C2, and WW domains bind to sites that are constitutively 'on'. Domains included in the survey are the most well-characterized domains and all data is obtained from the SMART domain database.

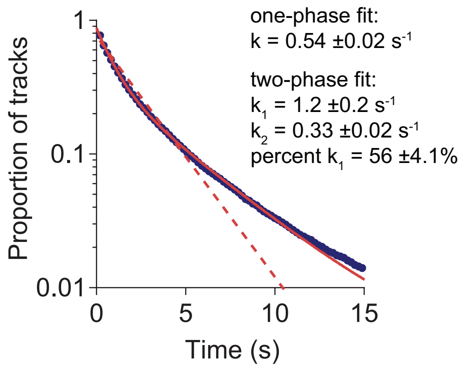

Figure S11: One- and two-phase exponential decay fits of ZAP70 tSH2-Halo Alexa647 binding to bivalent ITAM3. A two-phase decay model (solid red line) fit the track lifetime data (navy blue dots) significantly better than a one-phase decay (dashed red line). Average decay rates $\pm$ SEM are shown (n=9).

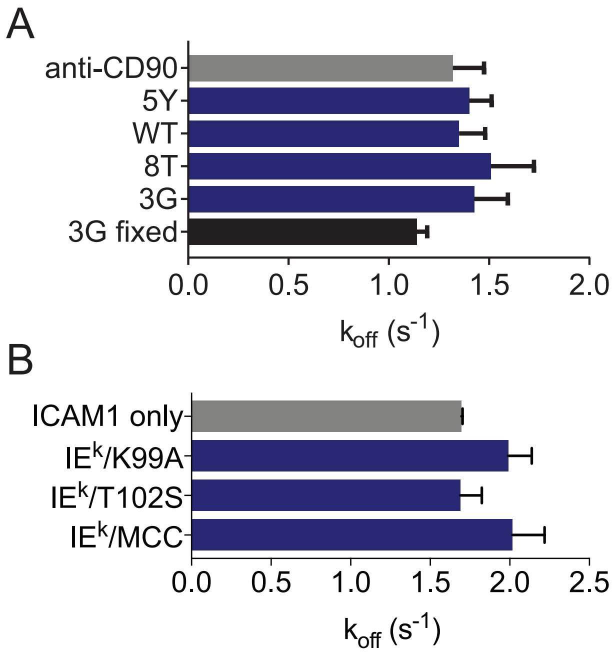

Figure S12: Fast phase decay rate component of fits from live cells SPT experiments. (A) Fast decay rates from fits of ZAP70 Halo-Alexa647 SPT data in ILA Jurkats stimulated with the indicated pMHC in live cells stimulated with the indicated pMHC or control (anti-CD90) or cells fixed after 5 min stimulation with 3G pMHC (3G fixed). Corresponding slow phase rates are shown in Fig 5E. (B) Fast decay rates from fits of ZAP70-GFP SPT data in primary mouse AND T cells stimulated with the indicated pMHC. Corresponding slow phase rates are shown in Fig 5I. Data are from at least 8 cells per condition imaged in three separate experiments. Mean ±SEM are shown. There were no significant differences between conditions (one-way ANOVA with Tukeys post-test).

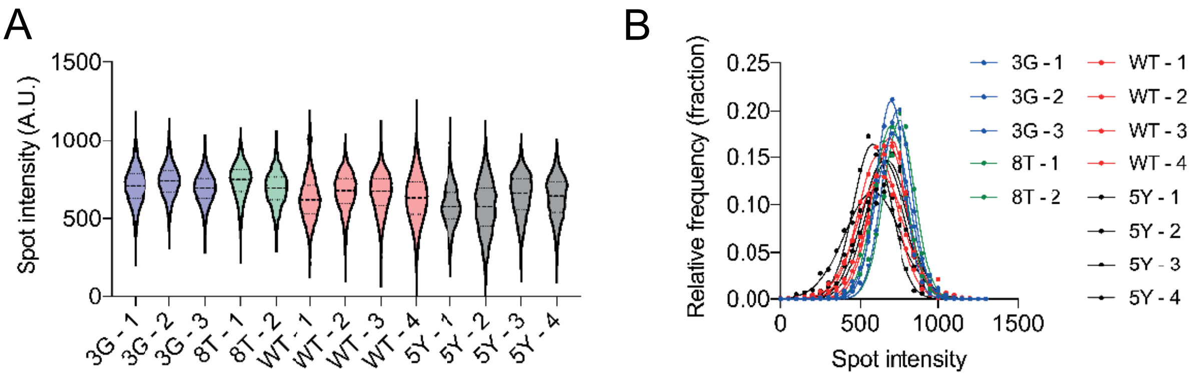

Figure S13: Spot intensities from single particle tracking. (A) Background subtracted spot intensities from image series of cells stimulated with the indicated pMHC. Data from individual cells in one representative experiment are shown. (B) Data in (A) plotted as overlapping histograms.
